# Bodywork as systemic and inter-enactive competence: participatory process management in Feldenkrais® Method and Zen Shiatsu

**DOI:** 10.3389/fpsyg.2014.01424

**Published:** 2015-01-12

**Authors:** Michael Kimmel, Christine Irran, Martin A. Luger

**Affiliations:** Department of Social and Cultural Anthropology, University of ViennaVienna, Austria

**Keywords:** bodywork, dynamic systems theory, systemic process management, synergies, embodied coregulation, enactive and extended cognition, expert skills

## Abstract

Feldenkrais and Shiatsu enable somatic learning through continuous tactile coupling, a real-time interpersonal dynamic unfolding in a safe dyadic sphere. The first part of our micro-ethnographic study draws on process vignettes and subjective theories to demonstrate how bodywork is infused with systemic sensitivities and awareness for non-linear process management. Expressed in dynamic systems parlance, both disciplines foster metastability, adaptivity, and self-organization in the client's somato-personal system by progressively reconfiguring systemic dispositions, i.e., an *attractor landscape*. Doing so requires a keen embodied apperception of hierarchies of somato-systemic order. Bodyworkers learn to explore these in their *eigen*function (joints, muscles, fascia), discriminate coordinative organization in small ensembles, and monitor large-scale dynamic interplay. The practitioner's “extended body” reaching forth into the client's through a resonance loop eventually becomes part of this. Within a bodywork session, practitioners modulate this hierarchical functional architecture. Their ability for sensorially staying apace of systemic emergence allows them to respond to minute changes and customize reactions in a zone of proximal development (*dynamic immediacy*). They stimulate the client's system with a mix of perturbing and stabilizing interventions that oscillate between *eigen*functions and their coordinative integration. Practical knowledge for “soft-assembling” non-linear synergies is crucial for this (cumulative local effects, high-level functions “slaving” the system, etc.). The paper's second part inventorizes the bodyworker's operative tool-box—micro-skills providing the wherewithal for context-intelligent intervention. Practitioners deploy “educated senses” and a repertoire of hands-on techniques (grips, stretches, etc.) against a backdrop of somatic habits (proper posture, muscle activation, gaze patterns, etc.). At this level, our study addresses a host of micro-skills through the lens of enactive cognitive science.

## Introduction

Experts in dyadic bodywork configure a rich set of skills in real-time to stimulate a client's self-organization (Blackburn, 2003; Myers, 2004; Tarr, 2008; Heller, 2012; Fogel, 2013; Porcino et al., 2013; Stötter et al., 2013; Stuart, 2013). Our contribution showcases, through a micro-ethnographic lens, how expert practitioner's in the bodywork disciplines Shiatsu and Feldenkrais® Method advance somato-personal development in the client through a process of “mutual incorporation” and how they make this a substrate for systemic sensitivities.

### Aims and overview

The first claim we submit is that while acquiring the requisite technical and sensory skills bodyworkers must also develop competencies we call “intrinsically systemic.” Practice constantly confronts them with the limitations of linear and mechanistic causality, forcing them to deal with dynamicity and complexity. Practitioners structure the interaction process and its dynamics in ways that are highly sensitive to how parts interrelate and dialectically interact with their embedding wholes, thus exhibiting a consistently holistic awareness. They are equally sensitive to the nature of non-linear synergies and take care to incrementally and gently nudge the client's dynamics rather than enforcing anything. These systemic sensitivities are partly explicated by metaphors, jargon, and conceptual frameworks of Shiatsu and Feldenkrais teachers, but partly remain pre-reflexive.

Our second aim is to demonstrate that a particular kind of situated embodied interaction with a profound interpersonal connection is affine to the systemic approach. We shall show how practitioners respond continuously to embodied emergence and hereby stay in a micro-zone of proximal development with the client.

The present work emerges from a cognitive micro-ethnography of interaction skills done with interviews and practitioner diaries. During this project the impression grew in us that the experts' perception of processes in our vignettes reflect what dynamic system theorists speak of. We discovered that Feldenkrais theorists like Carl Ginsburg and Marc Reese were greatly inspired by theories of dynamic self-organization and autopoiesis, including joint work with Esther Thelen, the renowned developmental systems researcher (Spencer et al., 2006). The first generation of Feldenkrais textbooks even took up key terms from cybernetics. Others such as Herfel and collaborators emphasize that Shiatsu theory itself contains essential aspects of dynamic systems thinking. We then found out that Günther Schiepek, Wolfgang Tschacher and others had developed a dynamic systems framework for describing non-linear processes in psychotherapy. All this inspired us to explore systemic skills in our context, which is “as embodied as it gets.” We also felt that merging this with tools from enactive cognitive science, motor control theory, and ecological psychology provides flesh and blood to the concrete implementation of the process.

Our qualitatively rich micro-genetic descriptions of bodywork sessions brought to the fore embodied micro-repertoires and situated strategies which proved to reflect process management with a holistic and systemic logic to it, thus converging with the dynamic-cum-systemic key ideas manifest in bodyworkers' descriptions of their trade. To explicate bodywork skills from a comparative distance we presently adopt the terminology of dynamic systems theory (DST), without relying on its mathematical tools. In keeping with clinical applications to human psychology (Tschacher and Dauwalder, 2003; Strunk and Schiepek, 2006) we aim at a DST and *synergetics* based metatheory for ordering processes and process management (Haken and Schiepek, 2010; Schiepek et al., 2013). Although several of our claims necessarily remain hypotheses we wish to contribute to the nascent dialog with practitioners who employ DST parlance by investigating “how changes at the micro-level of relationships between the system's constituents give rise to new patterns of behavior at macro-levels” and how “constituents of a system act together to constrain the multiple actions of other constituents” (Lavelli et al., 2008, p. 45). The specific kind of viewpoint we shall develop here follows Fogel's qualitative model, which is based on informational dynamics and which he contrasts with quantifying, measurement based viewpoints (2006: 26):

“Qualitative dynamic systems research is ideal for translational applications. Models that are expressed in terms of statistical interactions between quantitative variables are probabilistic and often far removed from the everyday process of meaning making as a social system. New models and interventions that rely on an understanding of the informational dynamics of the change process could be immediately applied to the work of practitioners and participants because these models are expressed in terms of the meanings that are already present in the system.”

Practically speaking, qualitative DST parlance is a crystallizing core for the comparative study of bodywork skills that future researchers may find useful. In our view, this essentially 3rd person process theory can connect to 1st person correlates, i.e., practitioner's strategies of a highly sensorially “grounded” nature. What follows is a plea for groundwork on the micro-interactions underlying the systemic process, i.e., for exploring how systemic thought in the abstract is implemented in concrete somatic interaction.

### Disciplinary background and goals

Founded by the physicist and judo teacher Moshé Feldenkrais (1904-1984) after the 1940s, the Feldenkrais Method® views itself as a somatic educational system for enhancing the body image, heightening awareness and expanding one's movement repertoire (Feldenkrais, 1993, 2005; Russell, 2004; Ginsburg, 2010). Two different styles may be contrasted. Awareness through movement (ATM) lessons are presented verbally. The practitioner guides several participants through a series of movements. The participants are invited to discover effortless and pleasurable exertion, while increasing their musculoskeletal awareness and spatial orientation (cf. Connors et al., 2010). The aim is to help them realize new proprioceptive and kinesthetic possibilities. In Functional Integration (FI)—our present focus—the practitioner manually guides a single client's movement. Feldenkrais himself characterized this tactile communication process as “two nervous systems dancing together.” Feldenkrais practitioners work through a skeletal and neuromuscular interface—while certainly not narrowing the intended outcomes to this. In terms of techniques FI includes a variety of small repetitive mobilizations, left-right mirroring, as well as differentiation and integration of movement patterns. Sensorimotor differentiation is thought to arise through minimal stimulus differences and thereby tunes the nervous system (Rywerant, 2003: *cybernetic-kinesthetic model*).

Shiatsu, here with a focus on Zen Shiatsu after Shizuto Masunaga, employs manual techniques and attentive touch to harmonize the client's *Ki*-system^1^. *Ki* (Japanese for *Qi*) is conceptualized as an encompassing “lifeforce” that sustains and coordinates various functions of the body-mind whole. Shiatsu practitioners report that they develop a “sense” for *Ki* in their practical training, thus making “energetic” states amenable to modulation. This scientifically scarcely theorized medium (Oschman, 2000) is phenomenologically acutely real, while appearing more elusive to the layperson than musculoskeletal categories. The *Ki*-system is deemed an energy supplying resource, an informational vehicle, and a regulatory mechanism surpassing the central nervous system. Within this system, Eastern Traditional Medicine discerns *Functional circuits* that cut-across various dimensions of the body-mind whole. This *Circuit* model incorporates centuries of systematically observed correlations between *Ki*-patterns on the one hand and physical, cognitive and psychological conditions on the other (Masunaga, 1987; Beresford-Cooke, 2011; Reder, 2013). This model only partly corresponds to Western biomedical and psychological concepts. It constitutes a framework to interpret collected indicators (via anamnesis, observation and palpation) and deduce from these which *Functional circuits* are presently dominant or diminished in the overall *Ki* distribution. Shiatsu practitioners aim to foster the free circulation and even distribution of *Ki* that guarantees musculoskeletal mobility, metabolic activity, nourishment of tissue, and mental and emotional balance. After diagnosing the client's momentary *Ki* distribution practitioners apply impulses for stimulating, collecting or scattering *Ki*, e.g., by stimulation of acupressure points (*Tsubos*) and *Meridians*^2^. All manual techniques affecting musculoskeletal mobility, fascial stress release, etc. additionally benefit unrestricted *Ki* circulation.

When we juxtapose the two practices, the manual techniques and indeed the dominant “medium” differ: One skill focuses on movement, the other on subtle energy. However, FI and Shiatsu have many operative structures and the goal of *somatic-sensory pedagogy* in common:

- Both obey the systemic metaphor of homeostatic “dynamic balance” (i.e., *metastability*).
- Both furnish springboards for the client's self-regulating capabilities. Practitioners awaken existing resources in the client or jointly exploit new ones.
- Both purport to widen the action repertoire, advance adaptability, and strengthen resilience in a process of sensitization. This happens by de-habitualizing familiar locomotory, muscular, and energy management patterns.
- For many clients the aims reach beyond ailments, into the affective, mental, psychic (Hanna, 1988, 2003; Posadzki et al., 2010), and even *ecosomatic* realms (Burns, 2012). A transformation of life habits and the personality is at issue.
- The embodied dialog works via a somatic interface, occasionally supplemented by speech.

Both disciplines summon wide expanses of expertise, with profound mastery being reached after a decade or more. Apprenticeship in Feldenkrais and Shiatsu shapes a “knowing body” (Section “Embodied Skills”): It transforms the hands, eyes, and other senses to yield extraordinary somato-cognitive resources. In the moment-to-moment interaction this enables the practitioner to draw on a variety of enactive micro-skills such as “smart” perception, dynamic sensory disambiguation, basic motor routines (“grips” for mobilizing joints, etc.) and task-conducive imagery, all against a backdrop of postural and sensory pre-calibrations.

### Somatic coregulation

FI and Shiatsu cultivate “the art of encounter” between a trained giver and a layperson client. High quality personal coupling and mindfulness is essential (Blackburn and Price, 2007; Nolan, 2014). Rapport is both a means and a goal, for it engenders trust, a cooperative attitude and, hence, compliance. A well constituted “dyadic bubble” establishes resonance loops for perception and action that allow a very fine dynamic attunement between the two bodies.

Generally, bodywork exemplifies *participatory sense-making* (Fuchs and De Jaegher, 2009). The two bodies are “mutually dynamically entangled” (Froese and Fuchs, 2012) in a bi-directional feedback loop and respond to each other continuously. Irrespective of the client's superficial inactivity, subtle somatic signals make for active engagement. A genuinely two-way street with ceaseless reciprocal causation arises, a condition variously dubbed *coregulation* (Fogel, 1993, 2006) and *inter-enaction* (Torrance and Froese, 2011). Froese and Fuchs (2012, p. 212) specify that both individuals' intra-bodily resonance loops (affective feedback, sensation, etc.) are embedded in an interpersonal resonance loop and an inter-affective system^3^. This “union of two nervous systems” (Stuart, 2013) goes with a subjective feel of strong rapport and genuine *interbeing*. Thus, practitioners report a salient perceptual shift when two individual systems become a genuine dyad, which is frequently described as boundaries to the client becoming fluid. A quote from Moshé Feldenkrais expresses this idea:

“Through touch, two persons, the toucher and the touched, can become a new ensemble: two bodies when connected by two arms and hands are a new entity. These hands sense at the same time as they direct. Both the touched and the toucher feel what they sense through the connecting hands, even if they do not understand and do not know what is being done” (Ginsburg, 2010, p. 267).

From a 3rd person viewpoint, coregulation manifests a superindividual dynamic with a degree of its own autonomy. We can thus seek “to understand how the patterning of the collective is related to the coregulation between the constituents” (Fogel, 2006, p. 8). This opens the interesting possibility of studying the coordinative process dynamics (cf. Haken and Schiepek, 2010) and, from our angle, invites asking how bodywork practitioners, who experience coregulative process signatures, learn to expertly read and shape these^4^.

In terms of enskilment for coregulation two things bodyworkers need to master are (a) attuning with the client to establish resonance and (b) reading signals of the other body and acting on them in real-time. Accordingly, bodyworkers train capacities for tactile sensing and subtle stimulation. The ability to take up the client's “offerings,” i.e., *affordances* (Gibson, 1979), at each moment is paramount. Action guidance comes from tactile, kinesthetic, and visual information from the other body. Practitioners in return offer opportunities to the client.

Another skill to learn is to literally extend into the other body to widen control beyond the tactile interface. Remote body parts can not only be sensed, but guided through the resonance loop. An untouched part like the client's head can be targeted by mobilizing a leg. Frequently, the resulting remote feedback allows fine-tuned incremental control as the client's signals are passed on into the giver's sensorimotor control system. For this kind of remote control, practitioners create dyadic structures extending through both bodies such as a joint musculoskeletal chain, or a *Ki* resonance loop (body *extensions*, Clark, 2008; Froese and Fuchs, 2012).

Finally, while each bodywork encounter is unique, it is not that emergence in the client just “comes to pass.” Expertise means being able to continuously tweak and guide the process through strategies at different levels. The micro-genetic perspective embraced in the next two sections shall deal with systemic strategies (Section “Bodywork as Systemic Competence”) and their concrete embodied implementation (Section “Embodied Skills”).

## Bodywork as systemic competence

Shiatsu and FI furnish prime examples for *process management in (superindividual) systems* and for how systemic thinking translates into practice. Practitioners assist the client's somato-personal development. In this endeavor, the practitioners' acute awareness of complex systemic wholes and the frequently non-linear synergies between the parts is a precondition for managing somatic emergence. They routinely deal with structural signatures of dynamic systems, including *attractors, emergence, dynamic stability, non-linearity, circular causality, multi-causality, etc*. A typical practitioner thinks and acts systemically, at least implicitly. Accordingly, Feldenkrais practice sees complaints like psychosomatic pain as a non-linear dynamic involving perceptual, biomechanical, neural, emotional and other elements (Russell, 2003; Ginsburg, 2010). Shiatsu even has its own process theory, the *Driving and Damping Cycles* of the *Five Phases* model (Herfel et al., 2007).

### General aims and structure of synergetic process management

The clients' resilience and process competence depends on a dynamically stable, harmonious interplay of systemic functions and elements. Bodywork fosters a heightened sense of somatic self-efficacy by sensitizing the client at multiple levels, including intrapersonal, interpersonal, and ecological ones. Expressed in DST parlance, the systemic configuration and *intrinsic dynamics* is reconfigured: from relative disorder to order (cf. Tschacher and Grawe, 1996; Tschacher et al., 2007), from rigidity to greater adaptability, or from dysfunctional order to a salutary alternative when clients' systems are caught in a stable, yet dysfunctional *attractor regime* (Section “A Meta-Theoretical Description”).

Let us first survey how bodyworkers deal with complexity through *synergetic* management principles. They emphasize that emergence must be respected and immediacy is of paramount importance. Therefore, they frequently seek optima among flexibly activated principles and *soft-assemble* the process via a situated coordination of elements (Thelen and Smith, 1994; Kelso, 1995; Kello and Van Orden, 2009), rather than using hard-and-fast routines. Bodyworkers regularly intersperse improvised phases and adapt best practices dynamically, even if some (sketchy) strategic planning may occur. Any such plans are persistently modulated or re-evaluated in accordance with momentary feedback from the client.

Throughout the whole process, bodyworkers respect general maxims of systemic intervention that Haken and Schiepek (2010) call *generic principles*. These transversally infuse the process:

- Practitioners know that sustainable somatic learning depends on stable conditions. Destabilization in a context of stability is crucial (ibid.: 437).
- Practitioners configure a safe space of trust through a non-judgmental, accepting atmosphere as well as attentive support.
- An attitude of “requesting” and “making suggestions and offers,” verbally as well as in tactile intent, engages the clients in a co-equal process instead of making them passively “handled” recipients. Practitioners present changes as painlessly and pleasurably as possible. These precepts foster receptivity, motivation and self-responsibility on the part of the client.
- In order to heighten the client's motivation and self-efficacy, stimulation must be meaningful and timely (*kairos* principle). Systems can only permanently assimilate order which is somatically meaningful. Reorganization needs to be coherent with the self-image, needs, and extant capacities.
- All inputs are suggested in a sensitive, yet precise way to convey a maximum of information. The idea is to “advertise” favorable patterns via their sensory effect. Although clients may temporarily relapse into their habitual patterns, order reached earlier—especially of gratifying states—is somatically remembered and can be re-triggered (ibid.: 245). For similar reasons, system history is always factored into the treatment.

Interacting without synergetic sensitivities has significant disadvantages: “Pre-fabricated” recipes not only preclude adapting to a person's unique dynamics or unexpected changes, but can altogether overlook the client's priorities. A non-systemic view can lead to a merely symptom-oriented treatment bypassing the clients' deeper needs or making them swing back to a detrimental attractor if the wider systemic disposition has not changed.

**Figure 1:**
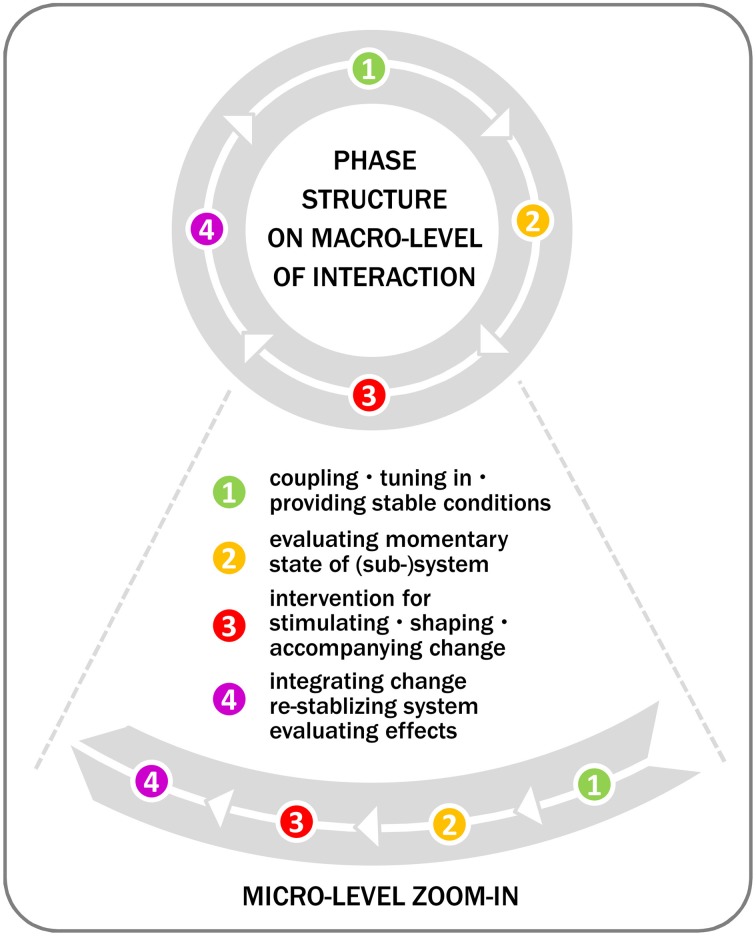
**Phase structure**. As the intervention phase indicates, multiple micro-level sequences may be nested in the global process and fractally replicate the macroscopic in the microscopic structure. In certain cases, these phases are condensed or may even temporally coincide, e.g., when intervention simultaneously creates stable conditions, elicits information, and provides impulses.

In its global structure, a typical bodywork session lasts 45–70 min. Each stage consists of multiple local interventions that are embedded in a wider strategy tailored to the client's needs. A degree of *fractality* meets the eye here. For example, practitioners create stable conditions and run an evaluation both at the beginning of the session and when starting a new sub-routine. Figure 1 depicts this sort of phase structure with micro-loops embedded in the intervention phase.

**Figure 2:**
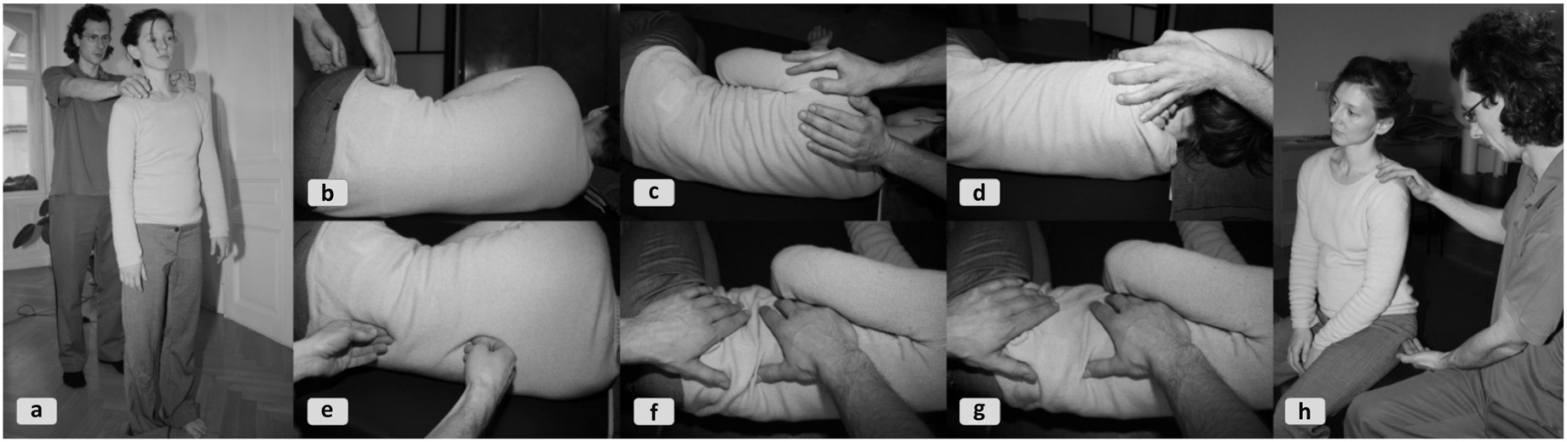
**Selected snapshots from an FI session**. **(2a)** Visual and tactile anamnesis of body part interplay. **(2b)** The responsiveness of the pelvis in relation to the upper body is explored (“cardinal directions of the pelvis”). **(2c)** The shoulder is differentially moved in relation to the chest (“cardinal directions of the shoulder”). **(2d)** Jointly moving head and shoulder, with their distance kept stable through fixation, in relation to the lower chest and spine to stimulate responses in the latter. **(2e)** Tactile probing allows the practitioner to test the vertebrae's *eigen*-tendency in the lower thoracic spine. Two points are touched to stimulate tiny side-bending motions of the vertebral joints. The attentional focus lies on releasing the erector spinae muscles and on establishing pelvis-chest communication via the spine (“elongating the spine”). Next, starting from the neighboring vertebrae, the proximal synergetic interplay is explored. **(2f+2g)** A gentle combined stimulation of the pelvis and the chest alternates between approaching, receding and random movements (“harmonica-like oscillations”) and widens both the diaphragm and the lower belly for deeper breathing. **(2h)** In the integration phase the full task is simulated in sitting position.

The following FI vignette (Figure 2) illustrates how the practitioner evaluates the somatic system, tailors a sketchy treatment strategy, fleshes it out it with responsiveness to situated affordances, and helps the client integrate new patterns in a familiar task-context.

- **Tuning-in/anamnesis**. The client who complains of stress-related neck pains would like to sit more effortlessly during computer work. Starting from this, the practitioner visually gauges how differentiated the interplay of the client's body regions is during walking, standing or sitting (**2a**). The trunk moving en bloc, particularly in the ribcage and shoulder girdle, suggests undifferentiatedness. Pelvis and head movements confirm this impression. Next, the practitioner establishes tactile contact and attunes with how the client's muscle tone is distributed. The purpose is to probe habitualized “attractors” (Section “A Meta-Theoretical Description”), to identify relevant sub-systems and their patterns of interplay. For a more specific exploration, the client comfortably lies supine on the Feldenkrais table with bent knees and feet flat. The practitioner applies a finely adapted thrust on the ischial tuberosity with the index finger enclosing the thumb. The resulting sense of direct bony contact allows him to probe into and thus address the whole skeletal configuration. He now sends gentle force impulses via the pelvis toward the head, while monitoring how well these proliferate along the spine. The force appears noticeably blocked, smothered, or diverted in the lower thoracic spine. This confirms the original impression of local non-differentiation and further directs attention to the lower ribcage area.
- **Strategy choice**. An FI lesson for *sitting and standing functions* in a pain-reduced side-lying position is selected, featuring the leitmotifs of *side-bending* and *differentiation of shoulder, ribcage and head*. The guiding notion is that distributing the excessive tone in the neck's wider surroundings will foster systemic balance in keeping with the client's needs. This sketchy plan remains to be fleshed out in detail along the way.
- **Intervention**. Exploratory variations are introduced with the client lying in a stable lateral recumbent position (knees bent and head resting on a pad). The practitioner applies various manipulations (**2b–g**) to explore the potential degrees of freedom while repeatedly contrasting directions. Movement queries initiated from the head, shoulder, pelvis or leg (“preferred tendency of movement?”; “any alternative possibilities?”) release local inhibitions. The intensity of the movement queries is subtle but just strong enough to further stimulate the client's motor system. The idea is that her system will learn through supported self-agency (Reese, 1999). Many of these manipulations connect parts on this side of the body into a complex organic ensemble, as indicated by responsive and permeable functional chains (“Does the motion of single vertebrae involve the whole spine, sacrum, pelvis and head?”). The practitioner explores the synergetic interplay from various vantage points (“Does this element have to cope alone or can another one help? What if I inhibit the helper?”) and may intermittently spot-check if degrees of freedom in previously explored parts have changed. Finally, the client is rested again in supine position and is asked to inwardly observe recent changes. Then, for involving greater ranges of the body into the lateral interplay, similar interventions are repeated with the client lying on the other side. Lastly, the reference movement from the evaluation phase is taken up to test anew force transmission through the spine, its connectedness.
- **Integration**. The practitioner now integrates the movement pattern into a task simulating real-life situations such as sitting in front of a computer or comfortably grabbing something overhead to see, e.g., if side-bending can be recruited with ease and minimal impulses (**2h**). A miniaturized walking motion with the sit bones on the FI-table also fits this context. This is done with lengthened arms and then with arms dangling on the side. In closing, probing routines in standing and walking confirm the newly acquired range and quality of motion. These integrative techniques at the end of the session consolidate the gains before releasing the client into autonomy.

As a general point, this vignette highlights the constant switch of focus between global and local, of holistic sensorimotor patterns and their sub-functions. The somatic system is stimulated repeatedly from different angles to implicate an increasing number of functions. In FI, widening the possibility space through variations is believed to foster sensorimotor learning and flexibility.

### A meta-theoretical description

**Figure 3:**
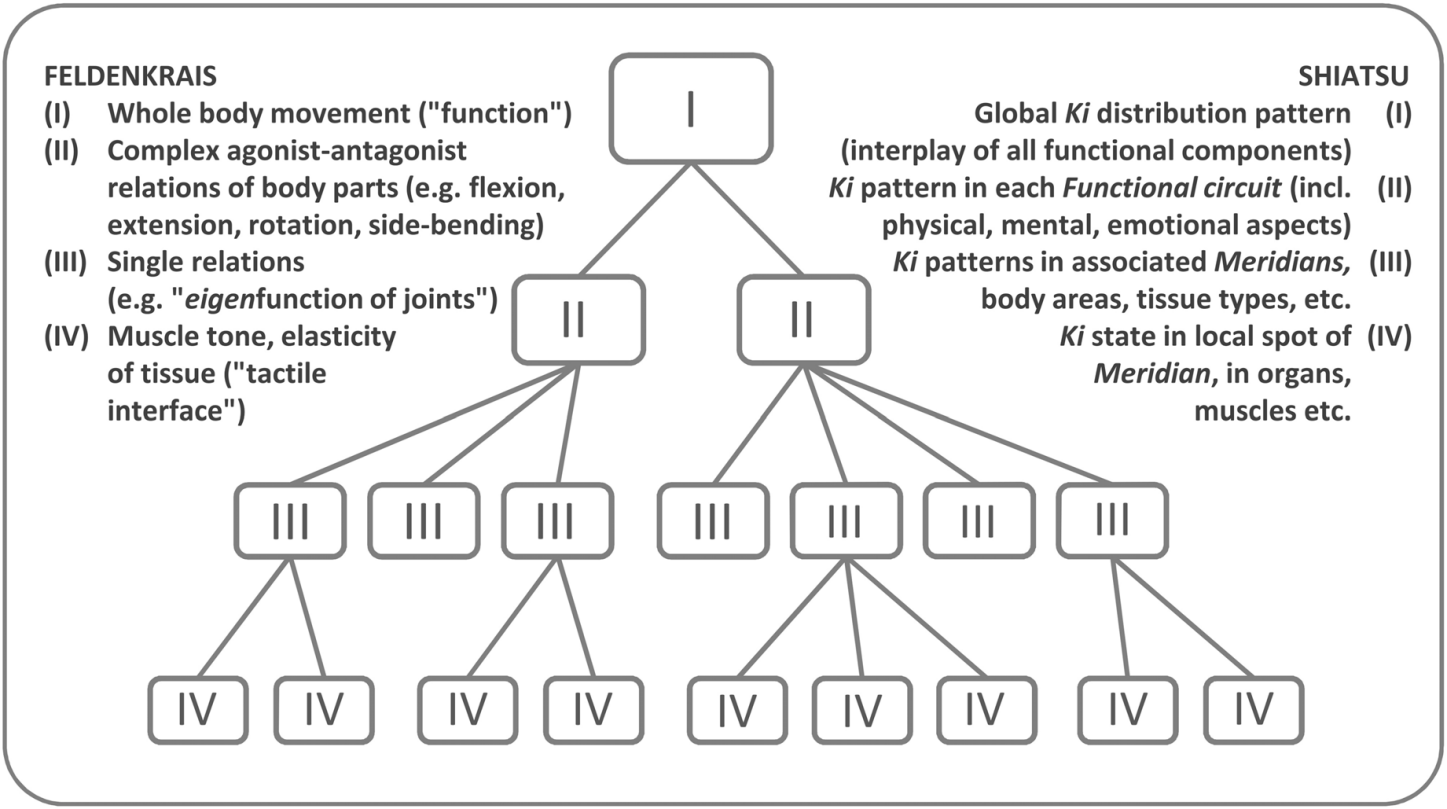
**The client's systemic order—a nested hierarchy from minimal- to highest-level functions and synergies**. Leaving the various situated system dynamics for later we illustrate here the *generic* architecture practitioners operate within. To take an FI example (see Shiatsu counterpart in the legend), the highest level corresponds to a complex body function like standing or walking. The second level contributes more restricted sub-functions or sub-circuits, typically of body segments like arms, legs, or spine. Such sub-functions in turn arise from synergies of yet lower-level components like joints and muscle groups. The third or fourth levels, respectively, constitute the lowest meaningful layers and define the maximum degree of resolution (Schiepek et al., 1992, p. 241). More subordinate synergies down to the molecular level lie out of scope.

We may now, for our purposes of theoretical bridge-building, relate bodyworkers' process management competencies to DST concepts before we demonstrate their subjective, sensory correlates. Basically, practitioners perceive the client's body as a complex system with nested levels of ordered functional sub-systems. The DST notion of *order parameters*, i.e., collective variables of somatic organization, manifests in musculoskeletal and energetic phenomena “that matter” to the disciplinary logic^5^. Specifically, the client's system is stimulated based on its conceptualization as hierarchically nested levels of order that cooperate, compete, or co-exist (Figure 3). In keeping with DST, embodied transformations of order occur via greater or lesser *phase shifts* (Thelen and Smith, 1994; cf. Buchanan and Ulrich, 2001), often in sudden leaps. Also, when multi-component systems shift to a new state, changes in the components may suspend higher-level patterns until a stable re-adaptation emerges. Changes are thought to run through a progression from rigidity, via acquaintance with novel organization, to full familiarity (Bernstein, 1967).

The order hierarchy, at any given point in time, concretizes as an *attractor landscape* (Haken and Schiepek, 2010, p. 340) representing states and routines into which the client easily “slips.” Bodywork is about effecting a gestalt change in this landscape by transforming its valleys and peaks. Some landscapes are more resilient and prone to adaptive self-organization than others. Experienced practitioners must therefore possess a refined sensitivity for both, dysfunctional and functional attractors. They recognize tipping points and input-prone moments and know how to channel extant dynamics, when to confirm, reduce, change, dynamize, or otherwise modulate them, when to take the lead and when to pace along. Practitioners thus *manage* transformations of the landscape by a stream of modulations that target either individual attractors or their relationship. Depending on the context, interventions can aim to:

- flatten out a particular attractor trough,
- confirm or deepen the attractor,
- calm excessive fluctuations,
- shift toward a more beneficial attractor to re-stabilize there.

To avoid deepening a dysfunctional attractor one can lead away from it or strengthen neighboring ones. Sometimes, jointly exploring tipping points suffices to boost the client's self-confidence with and grasp of the zone. Practitioners aim at conditions for risk-reduced phase shifts to new attractors or attractor regimes (i.e., by shifting the weights among attractors). Within the zone of “beneficial patterns” a diverse attractor landscape is advantageous, e.g., having multiple options of movement control or *Ki*-organization. When practitioners introduce new options to experience this lessens the likelihood of a few “domineering” attractors and thus of stereotypical responses (Haken and Schiepek, 2010, p. 426). On the other hand, systems displaying dysfunctional hyperflexibility or overburdening parallel tasks may stabilize by flattening some attractors and elevating others.

This intervention process is highly non-linear and cumulative. Over a treatment, bodyworkers will invariably mix (a) stabilizing with (b) perturbing stimuli. “Adjustment screws” on three dimensions are used to introduce and accompany the change process:

- *novelty:* new vs. familiar patterns (variation vs. redundancy and cyclic repetition).
- *diversity:* homogeneous vs. heterogeneous process gestalts (slowness / continuity / similarity vs. vacillation / accentuation / surprise).
- *dynamicity:* high vs. low impulse density, intensity, tempo, rhythmicity, accentuation, in-phase vs. anti-phase.

What makes an appropriate action strategy, however, is context dependent. A stabilizer in one context can become a perturber in another^6^. Practitioners need contextual awareness of whether an adjustment confirms, complements, or counteracts the client's ongoing dynamic. They carefully weigh in which states of order or transition to initiate action and how to combine stabilizing with perturbing stimuli.

### Probing the attractor landscape

A tangible, sensorial apperception of the client's hierarchic system architecture is crucial for bodyworkers. The reported complaint/request and the manifest somatic signals are diagnostically connected. By gradually probing the client's attractor landscape (i.e., the beaten paths vs. non-preferred states) practitioners (a) identify functional complexes presently relevant to self-organization and (b) determine parameters providing leverage. Despite differences in the somatic focus of Shiatsu and FI, the overarching heuristic principle is similar: Over- or under-represented elements indicate systemic imbalances (triggered either by *eigen*function disorders or lacking cooperation between parts). Both imbalances and balances have precise sensory correlates.

#### Coordination of components

In adaptive systems elements show a functional meso- and macroscopic interplay. This can be sensorially gauged by exploring how degrees of freedom in various loci co-vary when stimulated. Diagnosis seeks to spot over- or under-represented aspects and non-integrated subsystems in the holistic configuration. In FI motor functionality is tested, e.g., will a three-dimensional pelvis motion translate to neck motility? In Shiatsu a well-coordinated *Ki* distribution and smooth interplay of all *Functional circuits* is decisive.

#### Eigenvalue of local states

Adaptive order in a limb, joint, or *Meridian* should exhibit continuous responsiveness when addressed. Conversely, non-adaptive order will summon resistance, inertness, or deflection, e.g., when a force meeting a barrier perpendicularly veers off or “slips around” a particular area on the trajectory. FI practitioners check how unencumbered movements are and if they possess their full degrees of freedom. Adaptive movements are perceived as minutely responsive, “organic” and “mono-motivated” for the present task. Furthermore, a healthy system easily assumes a “neutral” state of action-readiness (*metastability*). In Shiatsu balanced *Ki*-activity correlates with effortlessly coordinated motion, well-nourished, elastic tissue and unrestricted *Ki*-flow. (Practitioners match this against the recognizable *Ki*-signatures of different *Functional circuits* as well as against the specific character of *Ki* in varying tissues, i.e., muscles, bones, ligaments, fasciae, etc.). The school of Zen Shiatsu, specifically, offers practitioners the following concepts for attractor recognition. Two dysfunctional types of sensory signatures are differentiated: states very high (*Jitsu)* and states very low (*Kyo*) in the local degree of (a) activity and (b) saturation of *Ki*.

#### Functionally isolated regions

In both disciplines local restrictions in breathing movement indicate isolated regions. FI hints include compromised erectness, lateral asymmetries in walking or in a resting position, body parts that smother or divert an impetus, and “blind spots” with so-called *sensorimotor amnesia* and disconnectedness. Analogously, in Shiatsu persistent *Kyo* areas lack energy or “forget” about their cooperative function. They may show signs of reduced metabolism and blood flow, lessened tone of tissue or loss of flexibility. They look pale and caved in, feel cooler, less animated or even stiff and brittle. *Kyos* react to touch with great neediness or—after drifting into stagnation—hesitantly, indifferently, with rigid resistance or nagging pain, and will even repel *Ki* channeled there and thus progressively exacerbate the lack. Complementarily, overactive *Jitsu* areas arise. They may display a reddish hue, heightened tone of tissue (bulging, overly firm, yet elastic) and respond to touch instantly with a resistant, definite, at times fierce feel. They may cause sharp pain especially when stagnant, a condition in which the *Jitsu* arrests *Ki*. Local *Ki* excess, painful tension, inflammations, and substance accumulation may follow suit.

#### Compensatory patterns

Basic compensation patterns are frequently recognized in diagnosis, but may also arise mid-way. In FI, when say the shoulder girdle is hypermobile a functionally connected part like the chest typically overcompensates with rigidity. In Shiatsu the abovementioned *Kyo-Jitsu* complementariness epitomizes this: When areas or functions lack *Ki* others compensate with over-activation or are worn down by trying to provide support. Even the entire system can resist change (to maintain a familiar pattern) when one sub-system is dynamized and another sub-system buffers this by way of reciprocal compensation.

#### Emergent process gestalts (Tschacher, 1997; Stern, 2010)

Bodyworkers also remain especially alert to self-organization in progress. They know the characteristic feel of continuous “response flow” of an adaptive system. Signal discontinuities can hint at fluctuations typical of transitory moments (*critical instabilities*),^7^ e.g., when muscle tone, heart- or breath rate vacillate. Instabilities will not alarm a practitioner as long as the risk of tilting to the detrimental side of two attractors can be counteracted (*symmetry breaking* toward the preferred side). In addition to fluctuations experts monitor critical values of strain, stiffness, hyperflexibility etc. in the client's system to mitigate detrimental dynamics.

### Intervention strategies

Bodyworkers often work on two or three tactile interfaces simultaneously by using hands, knees, elbows, and feet plus resistance from the support. Effects can extend considerably further into the functional complexes to which these interfaces provide access: Bodyworkers address effector spaces across the whole body. Thus, frequently stimulation generates remote effects, e.g., an FI practitioner can induce a full-body pattern like rotating the whole spine via a soft twist of the heel bone and thus “have the whole skeleton in his hand.” By using different depths and qualities of touch bodyworkers address different functional structures in a single area (e.g., fascial, skeletal, muscular, *Meridians*). Within the interface-effector space distinction stimulations can be more or less inclusive than the aimed at somatic order.

**Figure 4:**
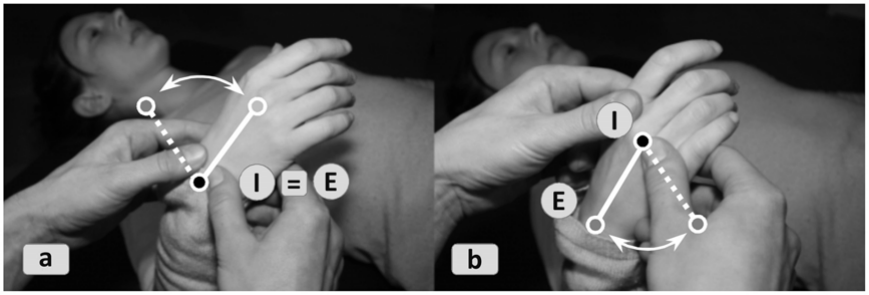
**Local vs. remote cueing**. Mobilization of the wrist joint can be effectuated **(4a)** locally by mobilizing the joint itself (i.e., *interface = effector space*) or **(4b)** remotely by inducing motion from the finger's base joints. While both interventions address the wrist's *eigen*function, they do so through different actuation pathways. The FI-principle of “proximal-distal-reversal” is applied here to let the client experience slight differences in the joint's and muscles' organization depending on where the movement is started from.

**Figure 5:**
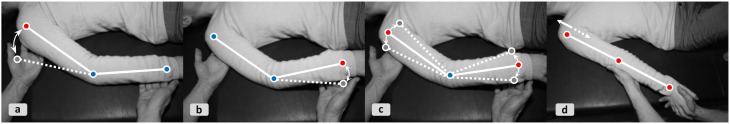
**Transforming the functional interplay in a limb**. (Red points signify motion and blue ones immobility relative to the surrounding space). The practitioner first addresses the subfunctions of the shoulder-elbow interplay **(5a)** and the elbow-wrist-interplay **(5b)** independently before introducing more complex arm flexion patterns though a co-mobilization of upper and lower arm **(5c)**. Differentiating variations are introduced, e.g., gentle to-and-fro movements in a see-saw fashion, starting with lifting the shoulder first and letting it sink when the wrist is lifted (and vice versa); bringing up shoulder and wrist simultaneously; synchronous mobilization with varying emphasis on both interfaces. In **(5d)** the whole ensemble is moved to simulate a pattern of the client's movement organization that requires the cooperation of all functional shoulder-arm components, as the pushing function addressed here.

In terms of relating spatial logic to systemic logic, three types of strategies can be discerned. In one recurrent strategy bodyworkers temporarily single out a functional component and optimize its *eigen*function, ultimately to improve its interplay with other functions, but with a local focus. A sub-synergy in the systemic whole is attentionally highlighted or even temporarily disconnected from other “players,” e.g., by fixation (Figures 4 and 5a,b). In Shiatsu the *Ki*-states of acupressure points (*Tsubos*) along a *Meridian* may be individually tuned and dis-inhibited to later allow for free *Ki*-flow through the entire pathway. In FI practitioners may mobilize single vertebrae, which later benefits equal worksharing among vertebrae. A variation is to address *eigen*functions remotely, as shown in Figure 4b.

The second possibility is to dynamize the relation between local functions through co-stimulation of two or more interfaces. This indirectly clarifies the *eigen*functions, while triggering the re-organization of the superordinate function(s). This optimizes the local functions' interplay, as shown in Figure 5c for an arm-flexion. The coordinative relations highlighted can either connect sub-synergies from the same hierarchical level to the higher-level patterns they partake of or cross-cut aspects across several hierarchical levels in the client's somatic order.

A third possibility is to directly stimulate a *complex* coordinative context. This higher-level functional ensemble is expected to impel embedded sub-synergies to re-adjust themselves, as illustrated in Figure 5d. In another FI example, practitioners simulate walking patterns with the client's leg (“artificial floor” exercise) to optimize the movement organization of the ankle joint and how the foot cooperates with the leg, pelvis, and upper body in locomotion.

Depending on context, bodyworkers will combine these three strategies differently. A frequent procedure in both disciplines is to treat a complex coordinative pattern in its holistic context, then disentangle it to transform selected aspects and finally reassemble it. This procedure can apply across the hierarchical order in a fractal fashion (i.e., practitioners may zoom into synergies at various nested levels). Figure 5 illustrates the stepwise integration of a local sub-synergy into increasingly complex patterns in the shoulder-arm system during an FI session.

### Synergetic process management

The discussed strategies require a profound understanding of coordinative functions and causal interdependencies in somatic systems. Practitioners recognize—and sensorially monitor—the make-up and genesis of *synergies*, i.e., coordinative structures realized in “functional groupings of structural elements (e.g., neurons, muscles, joints) that are temporarily constrained to act as a single coherent unit” (Kelso, 2009, p. 1537, cf. Latash et al., 2007; Turvey, 2007). To monitor this dynamic interplay, awareness of specific trade-offs and complementariness between local body states is crucial. A supportive practitioner must know how specific synergies become summarily constituted, including mutual neutralization, unidirectional damping, multiplication, or reciprocal compensation, and must consequently possess knowledge about the levels and nature of synergetic ordering (cf. Tschacher and Brunner, 1997).

**Figure 6:**
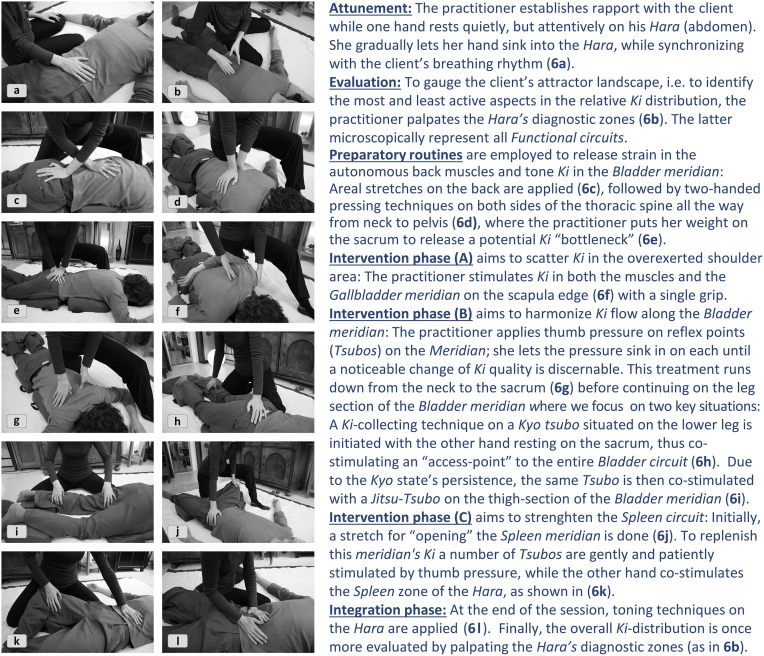
**A cascade of Shiatsu interventions**.

We shall analyze the following Shiatsu vignette reported by an experienced practitioner to showcase a complex synergetic strategy: By strategically probing and mapping the information on a conceptual backdrop (Section “Abstract Inferences”) the practitioner generates a complex systemic gestalt, which is progressively updated, drawn upon as a constraining backdrop, and later used for evaluating the outcome^8^. The practitioner chooses fitting interventions by and by, a cascade which brings forth synergies of increasing power for reshaping the attractor landscape. The example involves a client who is acutely overworked and suffers from painful tension between the shoulder blades. The practitioner seeks to release physical and psychological stress by working on anatomical structures and *Meridians* with largely extemporized, affordance-driven interventions—which are, however, fit into the earlier noted general structure of attunement, evaluation, intervention, integration, and final check (Figure 6). First, the practitioner applies empathetic, non-demanding touch (**6a**) to focalize the client's proprioception and encourage a relaxed parasympathetic mode (known to be favorable to self-organization). In return tactile feedback provides a first impression of the client's general *Ki*-state, which appears “vibrant but strained.” The standard *Hara* test (**6b**) provides a closer evaluation by localizing *Functional circuits* with excessive and lacking *Ki* activity. The specific check of the *Hara* zones indicates:

- excessive *Gallbladder Ki* (*Jitsu*);
- depleted *Spleen Ki* (*Kyo*);
- unstable and “flickering” *Ki*-quality in the *Bladder* zone (vacillating between *Jitsu* and *Kyo* indicators).

The practitioner now maps this sensorial input to Shiatsu concepts to infer the full picture (Section “Abstract Inferences”). To evaluate the current systemic status she must interpret the gathered sensorial data through the lens of the conceptual-diagnostic model in which manifest symptoms correlate with particular imbalanced *Ki*-patterns. Only after having mapped the troublemakers to *Ki*-logic can physical grips and techniques be selected that afford “navigating” the medium of *Ki*.

A sketchily defined procedure suggests itself from the default strategy of strengthening the weakest link of the *Ki*-system. Empowering the most underrepresented *Kyo* is assumed to result in a redistribution of *Ki*, which the client's system previously invested in the corresponding *Jitsu's* effort to compensate for the *Kyo's* deficiency. The practitioner also reckons that preparatory routines may be needed to open the *Kyo* for *Ki*-intake or to “detach” the excessive *Ki* from the corresponding *Jitsu* area if the latter cannot release it straight away. Implementing this weakest link strategy specifically suggests strengthening the depleted *Spleen's Ki*, which is in charge of mental labor, physical vigor, and psychological stability. Convergently, the *Gallbladder circuit's* strain has to be downtuned sufficiently to release the *Ki* “held hostage” there. Also, as the *Bladder Ki* is known to be crucial for systemic stress release and *Ki*-supply, the practitioner decides to balance out this subsystem as a further mediating factor for *Kyo-Jitsu* equalization. Therefore, the *Bladder meridian* is already factored into the preparatory routines for calming the nervous system and releasing tension (**6c**–**e**).

**Figure 7:**
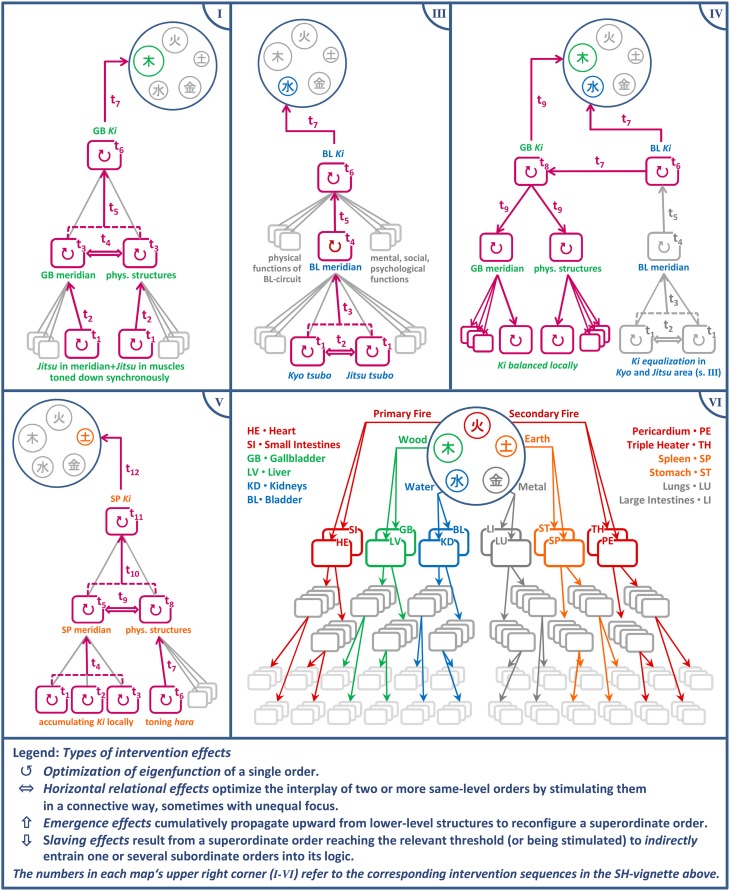
**Spreading activation: increasing upward propagation in the hierarchy and subsequent slaving downward**. The stepwise synergy build-up in the Shiatsu treatment (excluding the failed attempt II) illustrates several basic types of systemic effects. In each panel, the currently activated functions at the relevant point in time are displayed as highlighted sub-structures. The uppermost level depicts how the various *Functional circuits* feed into the compressing model of the *Five Phases*, which encapsulates the global *Ki* configuration (see panel VI and, for further details about the conceptual mapping the practitioner performs, Section “Abstract Inferences”).

We may now analyze the cumulative synergy build-up in systemic parlance. Each of the following intervention blocks is dedicated to one of the abovementioned strategic aims, with the means being selected in a more perceptually driven fashion from the repertoire while the process is underway. The bullet points with Roman numerals shall be illustrated in Figure 7, excluding step II (as the strategy remains without effect).

Aim A: **Setting free stagnant *Ki* in shoulder girdle and *Gallbladder meridian***

- **(I) “Local dis-inhibition and horizontal synergy for weakening dysfunctional attractor”**
  ***Strategy:*** The idea is to scatter the stagnant *Ki* in the muscles and *Meridian* structures of the shoulder area. Since they are not only functionally connected by their relation to the *Gallbladder circuit* but also topologically coextensive, the bodyworker can address them with a single grip (**6f**). It is immaterial which of the structures unblocks first, since the effect will usually spill over to the other aspect either way (horizontal synergy). This local dis-inhibition is expected to benefit the dissolution of the *Jitsu* in the entire *Gallbladder circuit*.
  ***Effect:*** The *Jitsu* is tangibly toned down and the strain between the shoulder blades thus reduced enough to release the client's tension. The preconditions are now in place to balance the *Bladder meridian's Ki* which should thereupon unleash its mediating power for the overall *Ki*-balance.

Aim B: **Equalizing vacillating *Bladder-Ki***

- **(II) “Relating isolated sub-synergy to superordinate level”**
  ***Strategy:*** While treating the *Meridian* the practitioner notices an acupressure point (*Tsubo*) with a conspicuous *Kyo* signature (dysfunctional attractor). To reintegrate the isolated *Tsubo* into its functional context the practitioner co-stimulates it with the sacrum (**6h**), a key access point to the *Bladder circuit*.
  ***Effect:*** No effect observable. Since the local *Kyo* attractor persistently resists adaptation and coordinated interplay an alternative strategy for optimizing *Ki*-flow in the *Bladder meridian* is selected opportunistically, as follows.
- **(III) “Optimizing component interplay to resolve dysfunctional attractor”**
  ***Strategy:*** The stagnant *Kyo* is co-stimulated with a *Jitsu* spot (**6i**) that was identified in passing as potentially corresponding to the former. The practitioner's assumption is that both local attractors might be part of a compensatory pattern inhibiting free *Ki* flow along the *Meridian*, as is confirmed next.
  ***Effect*:** After some moments of stasis, *Ki* begins to move along the “connective bridge” and dissolves the *Kyo* and *Jitsu* attractors simultaneously. After a certain point of equalization in the local *Ki*-distribution a tangible flush of *Ki* unfolds along the entire section of the *Meridian* (a phase shift in the local system). A moment later the effect dissipates through the whole system, thus entraining other sub-systems.
- **(IV) “Emergent higher-level synergy entraining subsynergies”**
  The client shows multiple signs of deeper relaxation, ostensibly due to the equalized *Bladder Ki's* calming effect on the autonomic nervous system, which reduces muscle tone throughout. The strong effect suggests that the changes in the *Bladder* and *Gallbladder circuits* have begun to transform the system. These changes in the global *Ki*-distribution now afford strengthening the system's weakest link of the *Spleen circuit* (i.e., the dominant *Kyo*), the session's main goal.

Aim C: **Balancing whole system by strengthening weakest link (*Spleen-circuit*)**

- **(V) “Elevating beneficial attractor by cumulative local effects”**
  ***Strategy:*** By stretching the *Meridian* (**6j**) the practitioner first seeks to make the “atrophied” leg section of the *Spleen meridian* amenable to *Ki* accumulation, which apparently lost a healthy capability to hold *Ki*. Then she applies *Ki*-collecting techniques to a number of *Tsubos* along the *Meridian* (**6k**). The practitioner finishes by toning the *Hara* (**6l**), a body area closely connected to the *Spleen circuit*, to further boost the *Circuit's Ki*-activity. Apart from this, the *Hara* technique was chosen for two surplus virtues in the given context: The *Hara* is a body zone connecting with all *Functional circuits*, which makes it inherently conducive to integrating the whole *Ki*-system, and the *Hara* focus redirects the client's attention from legs to torso, which in turn fosters centered somatic awareness.
  ***Effect:*** The improved *eigen*function and interplay of the *Tsubos* reinforces the *Ki*-activity in the whole *Meridian* and thereby benefits the entire *Functional circuit*. Together with the *Hara* techniques this cumulatively builds a beneficial attractor. A final test of the *Hara* zones shows the following advantageous changes of the global *Ki*-pattern.
- **(VI) “New equilibrium dissipates through system”**
  The *Bladder circuit* has been noticeably replenished and stabilized, resulting in a more equalized *Ki*-signature. The *Kyo* in the *Spleen* and the *Jitsu* in the *Gallbladder circuit* are still traceable, but reduced to an uncritical level. The system can be left to its self-regulatory devices from now on. The achieved local dis-inhibition and redistribution of *Ki* has apparently dissipated to all high-level *Circuits* and now downward propagates to all subordinate systems.

The stream of activation in the client's system, stimulated by the cascade of systemic intervention strategies, can be interpreted as gradual systemic activation spread (Figure 7). The vignette thus combines several strategic types afforded by a somato-systemic logic according to our earlier model. However, the client's system does not always respond to impulses as envisaged. Strategy II from the vignette illustrates this (which also explains why it is left out in the figure). When this occurs, the practitioner selects a new ad hoc route for synergy build-up. Thus, not only the ground-level operative tools, but even the mediating synergetic strategies are dynamically adjusted to the inevitably emergent nature of the interaction.

We may now wrap this up, both to add a few further items from the tool-box of synergetic process management and to clarify how important DST concepts relate to our discussion.

#### Modulating complex synergies

Frequently, bodyworkers inhibit or bracket out some co-synergetes to de-complexify the synergetic interplay. In FI practitioners may suppress reciprocal compensation or other trade-offs, e.g., by fixating other body parts. Alternatively, practitioners may facilitate a synergy with all its co-synergetes through specific aids. Reducing strength requirements is an example: Support against gravity shapes whether a nascent ability will manifest (cf. Thelen and Smith, 1994's work with treadmills for toddlers). Or, repositioning a person in the field of gravity induces a different experience of weight. In lying certain muscles will be relieved and liberate other resources. Conversely, in still other situations degrees of freedom may be deliberately opened, e.g., through labile supports, and the task's difficulty is progressively increased to trigger reorganization.

#### Stepwise synergy integration

In one basic transformational scheme an effect spreads out bottom-up. E.g., a newly established organization of the arm-shoulder movement propagates to the whole shoulder-girdle, ribcage, and spine. Practitioners frequently use intermediary states of relative order as springboards from which clients can make territorial gains. Frequently, functional semi-saturation suffices. The practitioner moves on before a local synergy is fully constituted. She is confident that with partial stimulation in multiple spots a connective synergy will emerge, as affine incipient pattern formations reach out toward each other and jointly amplify the local alterations. In the special case of *hierarchical synergies*, cumulative effects require respecting a particular sequential order. Elements simply need to be implemented with a key sub-synergy first to entrain the ensemble into further self-organization. The most elementary degrees of freedom must be sufficiently configured for others to join in an organized way. To illustrate, Feldenkrais practitioners commonly start with proximal foci before wandering outwards on the body: Basic movements of the spine/head (rotation, flexion, extension, torsion, side-bending) come first, followed by pelvic/shoulder girdle movements, etc.

#### Utilizing “slaving”

Another way of exploiting self-organization is to foster downward spreading activation. One route goes up and then down—as in the vignette—by working on lower levels for a while until an effect manifests in the macroscopic order. When the latter stabilizes it begins to downward propagate to the substrate and pull further aspects into its orbit (*circular causality*). As soon as elementary synergies connect, say, shoulders, ribcage, and cervical spine, previously unaffected elements will spontaneously join in, say, the head. In another scenario variant practitioners immediately target a higher-level order, e.g., in Shiatsu the action chosen at first contact may aim to trigger a parasympathetic reaction. Much as a chemical system exhibits greater reactiveness with rising temperature, inducing a mode of relaxation influences various sub-systems: more regular breathing and heart-rate, lower muscle tone and facial tension, greater receptiveness and attentiveness, *Ki* accessibility, etc. Such overarching levers come closest to a *control parameter*, i.e., a variable “to which the collective behavior of the system is sensitive and that moves the system through different collective states” (Thelen and Smith, 1994, p. 62) and which modulates the trade-off between elements by activating or dis-inhibiting resources (Haken and Schiepek, 2010, p. 438). However, such interventions must be thought of as non-deterministic, as each is assimilated, refracted, and transformed by the system before an effect unfolds (Strunk and Schiepek, 2006, p. 187).

#### Autopoiesis “kicks”

Oftentimes, brief stimulating or perturbing impulses induce self-organization. For example, particular Shiatsu acupressure points, e.g., *Gallbladder 21* or *Kidney 1*, foster self-regulatory dissipation of *Ki*. The mechanism here can be slaving, but also work via any of the other vertical or horizontal routes. Thus, in situations when a system is receptive an entire multi-step chain of spreading activation can be triggered through a single intervention, provided channels have previously been “lubricated.”

### How is systemic process management embodied?

We argued that bodyworkers are intrinsically systems thinkers. They possess an (implicit) understanding of the somato-systemic architecture and synergies unfolding within this framework. They frequently envision dynamic images of a (self-)organizing process, of how the elements interact to generate non-linear outcomes. Such images shape sketch planning and, as the session progresses, the evaluation of ongoing percepts and actions in terms of their effect on the client's system. Apparently, to keep track both of sensory occurrences in the here-and-now and their likely relevance in the bigger processual picture, many practitioners index the stream of embodied evaluations to a systemic framework. We might speak of mappings from experience to a conceptual “sketchpad.” Another possibility we cannot discount is that systemic skills implicitly arise without such conceptual mappings, simply by applying situated principles that add up to a good systemic effect. Thus, acting with systemic awareness minimally needs a collection of local strategies, but not necessarily an explicit ideology.

A distinctive feature of closely coupled embodied interaction as a means of process management is its *dynamic immediacy*. The process is kept hovering at a micro-zone of proximal development. Bodyworkers stay apace of every smallest dynamic increment and continuously respond to emergence with micro-actions by (a) amplifying input or modulating its quality, (b) changing the means when needed, (c) repairing problems, e.g., by boosting dwindling affordances, (d) buffering overshooting reactions and (e) optimizing boundary conditions underway. Whenever unexpected occurrences arise practitioners can immediately fine-tune and customize the ongoing process by virtue of their full embodied presence.

Dynamic immediacy is a prime precept for somatic learning, as it continuously challenges the client's proprioception and differential sensibility. Raising awareness for fine variations of actuation diversifies the client's potentials. Through constant perturbation and stabilization the client is kept hovering around the perceptual *limen*. This just-noticeable difference is sensitized (cf. Seitz and Watanabe, 2005). Dynamic immediacy is thus ideal for striking the important, but tenuous balance between blending in with the client's dynamics and cultivating a difference to it, e.g., by returning to familiar homebases and progressively exploring new things from there (Eilam and Golani, 1989).

Beyond the micro-dynamics, a further key to bodywork lies in how practitioners strategically re-allocate their attention over larger stretches of time. Their focus oscillates between parts and wholes with their various levels of interplay. A recursive to-and-fro movement follows logically from the non-mechanistic ethos of bodywork, which demands constant contextualization of local states. They accordingly allocate action in zoom-in/zoom-out fashion and entrain clients into this oscillation. An optimal systemic integration of micro-processes is hereby provided. Somatic learning thrives on the coequal stimulation of elementary *eigen*functions and their interrelations. Particulars become integrated in holons, the rationale being that components recursively confronted with coordinative functions optimally self-organize. Process management of this sort respects the multi-level nature of functional system architectures. In other words, emergence is addressed at multiple levels.

## Embodied skills

We now aim to ground the foregoing reflections, largely cast in systemic abstractions, in distinct embodied capabilities: Systemic sensitivities are implemented through a flexible tool-box of enactive micro-skills, many of which one might find in manuals or training curricula. During apprenticeship bodyworkers educate their senses to actively explore and elicit relevant perceptual information, but also to provide concrete interaction guidance, using modulatory feedback control, imagery, and related resources.

### Dynamic solutions

Many of our reflections fall squarely within enactive cognitive science (Di Paolo, 2005; Thompson, 2007; Froese and Gallagher, 2012), which claims that perception is active and that dynamic solutions are found by exploiting a continuous sensorimotor loop (instead of discrete “ready-mades”). How can one, by just blending into and tweaking an emergent dynamic succeed with a goal? Firstly, tasks may be openly specified, such that the means and sub-steps remain flexible. By analogy, scoring a soccer goal can not only be achieved in multiple ways, a forward is also free to abort the intention of scoring himself and to pass the ball. The same flexibility of intentions and means-goal relationship applies here. As one goes along intermediate goals and new means take on shape.

Secondly, in view of continuous interaction solutions need never be of a one-shot nature; in fact experts make the dynamic itself their confederate. Solutions are incremental and distributed over an extended time-course. We must think of them as an *arc* even if they last only seconds. In this arc, the expert's “closed loop interaction with the outside world” (Kirsh and Maglio, 1994, p. 542) incorporates attention-focusing, information gathering, generative, and full-out actions in various mixes. The micro-dynamic of bodywork mirrors the eye-tracking study by Ballard et al. (1997) who had experimental subjects recreate a pattern of building blocks from a template. The authors discovered that, while finding the solution, the gaze constantly flips to-and-fro between the template, the work-space, and the resource box. Many so-called epistemic actions are performed to yield further information, make the task manageable, and help think up matches (cf. Kirsh and Maglio, 1994). Tentative activity produces input and further dynamic adaptation. In bodywork, too, subjects refer back to the world itself multiple times to complete an action, rather than deciding at a single moment. Within this kind of enactive strategy the world becomes “its own best model” (Brooks, 1991; cf. Beer, 2003).

Thirdly, bodywork exploits genuinely higher-level properties of dynamic engagement (Auvray et al., 2009). That is, inter-enaction itself generates options unavailable to two passive bodies. The client's responses, however subtle, in conjunction with the practitioner's actions provide emergent affordances. An emergent self-stabilizing dynamic of mutual co-adjustments thus becomes the source of further options. E.g., the client's active breathing into a painful area while the practitioner mobilizes it typically generates a mutual pattern that clarifies percepts and presents new affordances.

### Enactive perception

Bodyworkers actively bring forth information (Noë, 2004). Sensing invariably requires (subtle) activity. E.g., resistance can only be probed by feeding impulses into the client's system. Bodyworkers thus *enact* percepts by *dynamic touch* (Turvey and Carello, 2011) or exploratory visual activity (O'Regan and Noë, 2001). They learn appropriate epistemic actions to gather and, by extension, stimulate information with, while also monitoring feedback from their pragmatic (overt) actions on the client. When actively inquiring into somatic responses, one particular trick is to subtly dynamize the client and thereby generate differentiated feedback. A refined *pacing* skill is associated with docking one's hands on the client's body in a way clear enough to feel and accompany movements, yet softly enough not to influence them. In fact, a desired sensation can be induced through “as if” interaction which aligns with the client's response anticipatorily (see *emulation*, Grush, 2004). In terms of resonance, applying this tactile quality “seduces” the client's system into providing clearer feedback. Emulation finely primes their perceptual apparatus to maximize chances of sensorially “clicking” with even minute traces of an activity pattern—which practitioners can amplify after having attuned with it. For example, they imagine putative micro-movements of the extremely subtle motility of inner organs like the liver or of small joints enveloped by muscles.

Frequently, bodyworkers simultaneously invest themselves with multiple epistemic strategies. To illustrate a typical cooperation pattern, the “*Mother hand*” in Shiatsu ensures a stable dyadic contact, provides a stabilizing context for change and keeps the global state in focus, while the “*Child hand*” works locally. In FI one hand often senses for effects at a distance of what the other does, so the two effector spaces are attentionally coupled and relationally interpreted.

#### Filters

Frequently, enactive pre-calibrations of the sensory apparatus are crucial. Depending on the situation, the senses are primed for a narrower or broader stimulus range. This creates perceptual filters of two sorts: Either one pre-configures the “hardware” of the sensory apparatus itself for a task, e.g., through a particular softness of touch or a particular gaze. Or, one pre-configures the attentional “software” by screening out irrelevant perceptual dimensions. Such calibrations can be very subtle. Expert practitioners can tune ostensibly identical acts of dynamic touch to selectively capture joint positions, muscle tone, tissue elasticity, or *Ki* flow. They do so by guiding their attentional focus to an (inner-)bodily target region or by attuning to qualitative dimensions like elasticity, *Ki*-permeability, temperature, vitality, or rigidity. Sensory filters may be pre-set for a whole task or they may dynamically change.

#### Sensory alertness

Experts equally know when to relax filters or active sensory exploration as such, i.e., when to just broadly register information with minimal activity of the sensorium. Even while momentarily using a filter the expert's senses have to be able to register contingencies in the background. Unexpected emergence requires multi-dimensional sensory alertness. To exemplify this, the attentional technique of “soft eyes” adopts a blurry focus for the widest possible angle of view and no visual fixations. Or, suppose a Shiatsu practitioner is exerting deep pressure on an acupressure point and suddenly the client's fascia shows an autonomic somatic reaction of “unwinding.” This calls for an instant switch by re-attuning the hands to a rather superficial, mainly lateral fascia movement and using decreasing pressure of the full palm (“*Butterfly touch*”).

#### “Smart” perception

Another way in which the educated senses of bodyworkers surpass ordinary capabilities is by employing “high-level perceptual functions” (Gobet, 1997; Gauthier et al., 2010; Goldstone et al., 2010). A major source of efficiency is smart perception where

“[t]he pickup function itself, rather than the subsequent processing, is the designated locus of perceptual sophistication” (Runeson et al., 2000, p. 533).

**Figure 8:**
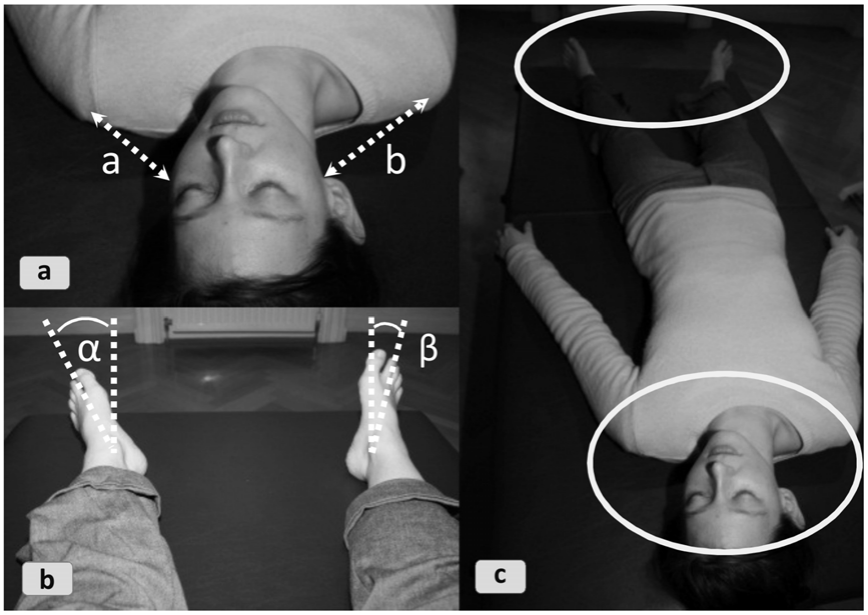
**Smart perception: two local perceptual gestalts inform a higher-level gestalt**. The practitioner picks from the perceptual field the local gestalt of the shoulder-ear relation **(8a)** as well as the local gestalt of the leg rotation **(8b)**. The geometric frame between the two loci is then perceived as meaningful integral relation **(8c)**. In skilled practitioners this can virtually happen at a single glance, but even if two glances are needed perceptual integration to a higher-level gestalt can ensue.

The gaze or hands are attuned to complex variables capturable with robust perceptual routines involving no inferential synthesis. The dynamic act of information pickup immediately compresses the perceptual field to “what counts,” frequently in terms of complex relational properties. In a typical example, FI experts attune their eyes to a walker's locomotory “wave patterns” wandering through the spine. They focus on the idealized body axis in walking and standing. A second example (Figure 8) for a smart exploratory technique in FI explicitly highlights the relational integration of inputs. The practitioner's gaze perceptually integrates an outward leg rotation (gestalt 1) and a head position with shorter ear-shoulder distance on the left side (gestalt 2). Both indicate a tendency toward leftward rotation in the spine. The gaze probes several spots, perceiving their relationship as a higher-level gestalt. Hence, smart perception task-specifically organizes the perceptual field. Setting “marking” anchors can bestow further smart organization to this perceptual field, as can using the appropriate patterns of dynamic attention management at the right time, e.g., figure/ground or perspectival operations such as zoom-in/zoom-out.

#### Dynamic specification

**Figure 9:**
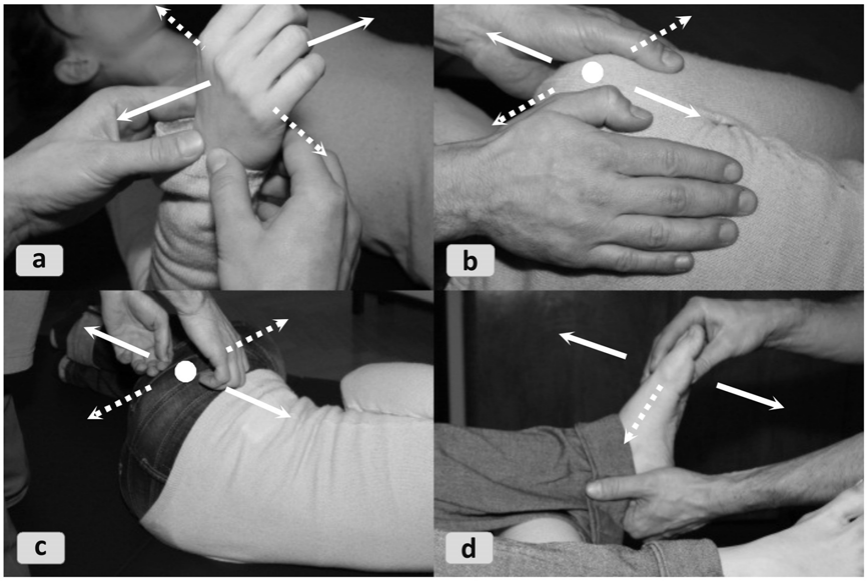
**Stepwise sensory discrimination by probing “cardinal directions” (FI)**. Gentle initial movements determine which micro-action patterns the area is amenable to and which it resists or finds unfamiliar. The dotted lines represent resistant directions and the solid lines yielding ones. Similar 3D movement patterns emerge around different parts of the body: **(9a)** wrist, **(9b)** shoulder blade, **(9c)** pelvis, **(9d)** ankle.

Perceptual exploration is temporally extended and structured even within a few seconds or less. Finely feedback-triggered micro-actions in response to a stream of micro-affordances lend relevant sub-structure to the smallest routines. To give Gibson's theory of affordances an appropriately dynamic format, we need to envision how practitioners realize integral actions such as tactile palpation techniques as a suite of many microscopic perception-action cycles. This process can unfold in two ways: In openly specified actions *sequential affordances* (Gaver, 1991) reveal themselves only step-by-step. Attention begins non-specifically attuned and the perceptual focus narrows while moving along. In many such situations experts simply trust that their epistemic and pragmatic micro-actions will produce enough further information to narrow down the possibility space by and by. This enactive strategy disambiguates means, strategies, and possibly even the proximal action goal itself in a *sensory funnel*, which progressively adds structural specifications while the task is underway. The problem solving exploits a to-and-fro motion between an incomplete action and proximal responses. To start the process, heuristics for exploratory sensory activity may be used, e.g., “probe the cardinal directions of a joint” and respond to micro-affordances by “start(ing) off with the easiest motion pathway” in FI (Figure 9).

Alternatively, routine techniques (Section “Action Skills”) may also possess phasic structure with internal causal and temporal dependencies. To respect these, experts rely on familiar *micro-affordance* sets (Kimmel, 2012). Thus, when initiating a multi-phasic technique, active perceptual exploration first allows the practitioner to anticipatorily “sense for” micro-affordances specifying the relevant onset threshold and later micro-affordances for continuation and micro-timing. Micro-affordances comprise specific feedback signatures—a particular elasticity, resistance, force, etc.—that trigger the action's next phase or allow modulating the movement direction or intensity underway. Notably, micro-affordances provide the appropriate coregulative sensitivity whenever the client's system responds gradually to the initial stimulus. Suppose a practitioner should intensify the impetus of a pressing technique only the moment the tissue softens or maximum elasticity is reached. In all situations when fluidity and quickness is of essence, bodywork experts anticipatorily prime their attention for such trigger signatures and the motor system for the incremental micro-action associated with each trigger.

Emergence is made a virtue of by incorporating dynamic feedback. Consider a Shiatsu technique for unblocking *Ki* flow by gradually increasing ribcage elasticity. Here the action is sketchily pre-specified, while fine-tuning and timing remain open. Muscular resistance determines how much pressure to apply and when to exert it. (Increasing the pressure in sync with the client's exhalation phase heightens the effect and makes it more agreeable for the client). Another point this illustrates is that micro-affordances may, when “go” signals remain absent, suggest a midway switch to alternative courses of action. E.g., a ribcage remaining rigid may suggest changing from vertical to a laterally swinging pressure. If this equally fails, the superordinate aim of unblocking *Ki* can be realized through totally different means like *Meridian* manipulation. Thus, whenever the desired feedback remains absent practitioners may intensify or change the stimulus, or even switch techniques.

### Action skills

**Figure 10:**
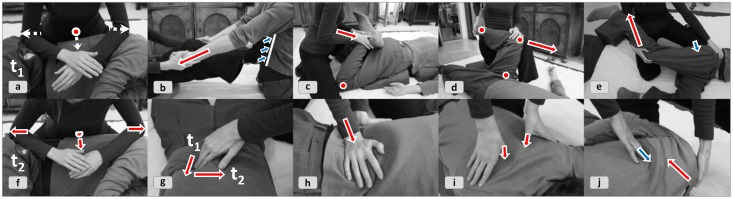
**Modular action concepts in Shiatsu**. The red vectors stand for the direction of pressure or pull, the blue vectors for simultaneously applied counterforce, and the dotted lines for preparatory actions in anticipation of the next moment. Various examples from practitioner's “toolbox” are illustrated. **(10a+f)** Transfer of body weight via elbows for an areal stretch. **(10b)** The feet provide a fulcrum for a shoulder stretch. **(10c+d)** Stretches of *Meridians*, muscles, tendons etc. combined with mobilization of joints. **(10e)** A large-scale *Meridian* stretch combined with acupressure through the knee. **(10g–i)** Attentive pressure, exerted by thumbs, fingertips, and palms, is intermittently combined with horizontal pull. **(10j)** Mobilization and pressure combined in rhythmic counter-motion for simultaneously moving *Ki* in the muscles and *Meridians* of the focal area.

In addition to *epistemic* actions bodyworker practitioners obviously need a repertoire of full-out *pragmatic* actions (Kirsh and Maglio, 1994). A set of basic micro-routines provides goal-directed motor programs for manual grips, stretches, presses, mobilizations, and so forth, i.e., “action-oriented representations” (Clark, 1997). Bodyworkers internalize this assemblage of micro-routines as ideomotor modules (Prinz, 1997; Koch et al., 2004; Shin et al., 2010) (Figure 10).

#### Action concepts

**Figure 11:**
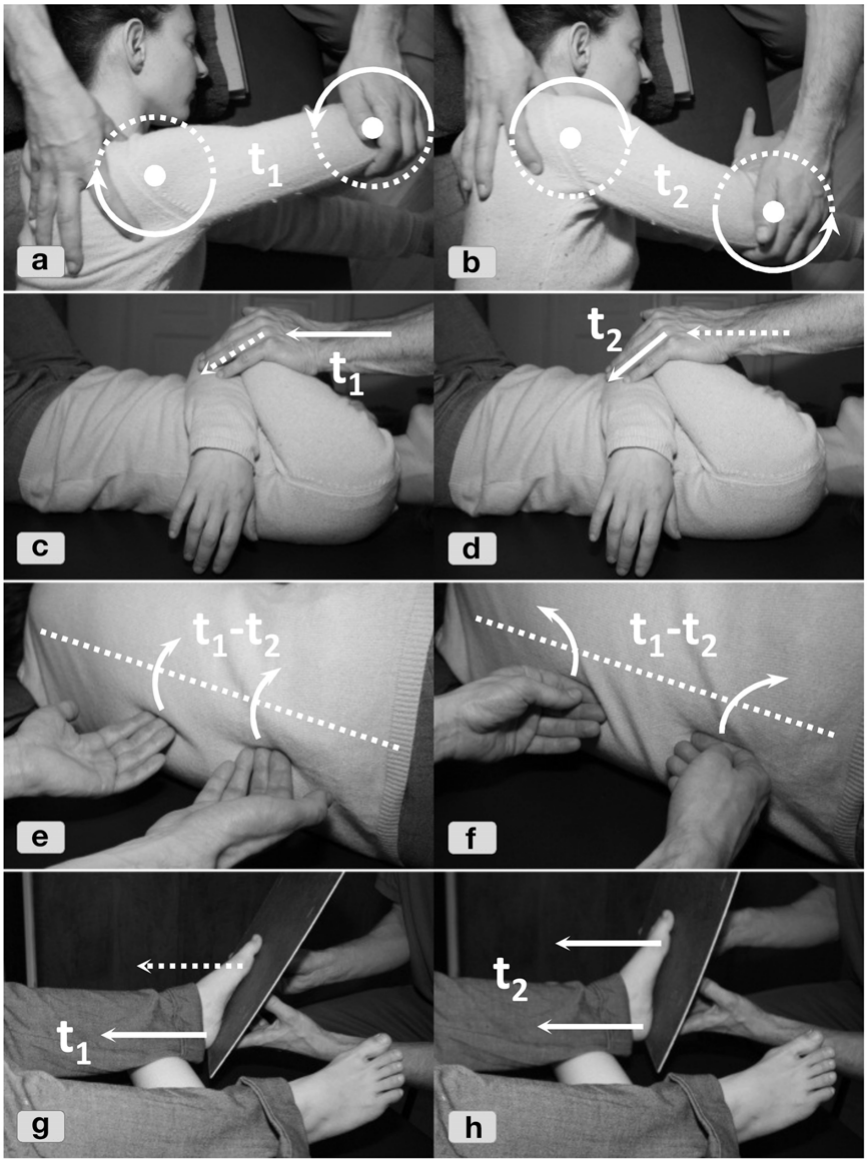
**Basic action concepts and multi-phasic gestalts in FI**. These can be thought of as modular interaction elements that can be repeatedly deployed with the appropriate contextual calibration. **(11a+b)** Figure-eight shaped curve gestalt in rotating the shoulder around the clavicle in relation to the elbow. **(11c+d)** In *t*_1_ the shoulder is addressed to initiate and deepen chest flection in *t*_2_. **(11e)** Touching the spine for rotation. **(11f)** Touching the spine for rotation & side-bending. **(11g+h)** “Artificial floor” simulation of walking pattern in supine position.

The term *basic action concept* refers to structured increments within complex and multiphasic ideomotor representations, as might be evident in the phases of a volleyball spike or ski jump that a sports expert represents (Schack, 2010). The Feldenkrais theorist Rywerant (2003) coins the term *manipulon* for such a modular action concept. Each short action, even at the temporal level of a single grip is, in the minds of experts, multi-phasic and contains fine-grained sub-structure represented as a string of minuscule *sequencing points* (Kimmel, 2012). Both the sequencing points and the motor gestalt they create—the whole “mini-clip”—are represented in terms of ideomotor imagery. Action concepts thus comprise hierarchically structured ideomotor gestalts, many of which may be stored as prototypes and can be further adapted for context-sensitive action variation (Figure 11).

#### Feedback control

While basic ideomotor units are first acquired independently of the client's or other real-time reactions it was suggested that bodyworkers combined these routines with feedback signatures for precise timing and fine-tuning of all sorts (*micro-affordances*). Instead of execution in “blind-flight” practitioners dynamically modulate micro-actions underway. By comparing actual to expected feedback they monitor and hereby correct against deviations the intensity, extension, direction, speed, phrasing, or accentuation of the ongoing activity (*forward models*, Wolpert et al., 2003, 2011; Pacherie, 2006). Further evidence suggests that experts use what we would call *enhanced* action concepts. These combine a spatio-temporal action image, the initializing motor commands, and expectations about accompanying tactile, visual, and proprioceptive feedback. Enhanced action concepts provide an integrative understanding of all sources of interaction control.

#### Imagery

In addition to full ideomotor commands of single-body techniques and small interaction scenarios, bodyworkers report using numerous types of schematic imagery: (a) *Global process gestalts* or *vitality contours* (Stern, 2010) help to envisage tempo, rhythm, and energy deployment over time, e.g., suddenness vs. gradualness, crescendo, ostinato. (b) *Image-schematic and force dynamic gestalts* (Johnson, 1987) include force attraction and repulsion, blockage, going with the force or against it, imagining a trajectory end-point, moving in intervals or a direct transition; countervectors such as a “rubber band” for stretches between two points, spiraling into a tissue, circling, vibrating into a point, snipping/snatching away energy, compression and torsion, stroking, “pushing a wave,” “see-saws”; complex trajectories like eights or 3-D shapes. (c) *Causal (“if-then”) imagery* of anatomical or physical principles like mechanic levers (Franklin, 1996) can be used to support action planning or to detect underlying principles while observing the client's body. (d) *Summarizing imagery aids*: Advanced experts project into space virtual points, vectors, axes, or shapes like a sphere representing the joint gravitational center. Such virtual gauges (cf. Hutchins, 1995) sum up the system's state for them while implicitly blending sensory data from multiple interfaces into an emergent organizing image. (e) *Node points*: When improvising, configurational images of “homebases,” i.e., junctures from which several continuations are known, provide usable end- and transition-points for the ongoing action and subserve a fluid connection (Kimmel, 2015). In sum, imagery is a crucial didactic asset and frequently couched in metaphors and key concepts (Kimmel, 2012). By being “introjected” (Kimmel, 2008, 2013) it organizes the body into the right synergies and activates the right effectors and sensors.

#### Control laws

**Figure 12:**
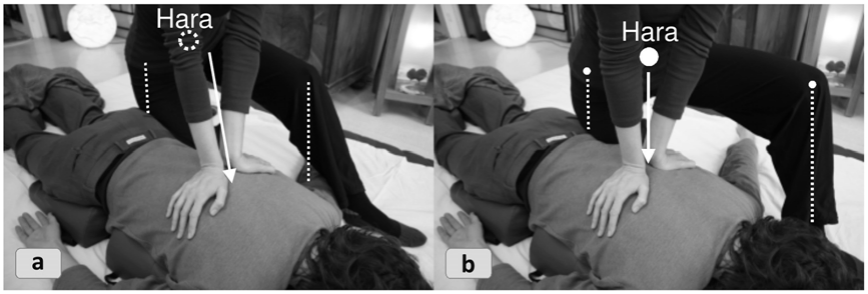
**Vector emerging from the *Hara* directed at the focal interface zone**. For illustrative purposes, we contrast a novice's rather ineffective approach to using the body weight **(12a)** with a proper vector emerging from the *Hara* **(12b)**. The proper technique directs the full center of gravity in the practitioner's lower belly onto the focal spot on the client. The practitioner is thus able to use compact force without exerting a lot of muscular pressure, while ensuring a maximum of inter-body rapport.

Building on feedback control mechanisms, *control laws* robustly link specific perceptual values to specific action responses (Warren, 2006, cf. *sensorimotor contingencies*, Noë, 2004). They are frequently expressed verbally in principles like “as long as tissue response is elastic you can go deeper” or “pursue the kinetic vector of least resistance.” When applying a control law bodyworkers monitor feedback in a particular dimension such as interpersonal distance, angle, balance, or pressure and gradually scale up their degree of action response after hitting a task-relevant onset threshold. Within the task, the applied control law specifies the relevant sensory dimension and *optimal* and *critical values* (Warren, 1984). The task can now be regulated in real-time via dynamic feedback. Deviations from the optimal value are corrected on-the-fly: the suboptimal parameter setting at time_(*n*)_ is adapted to conform with the optimal value at time_(*n*+1)_. Examples of this abound in bodywork: In passive mobilizations, which are used to familiarize FI clients with a new movement, it is all-important to support their incipient movements without losing the minimal cooperation of their motor system (for effective sensory pedagogy)^9^. For striking the proper balance, a control law specifies that the client's limb is just self-impelled enough when it remains in a closely defined range of tactile resistance. “Smooth” response continuity is felt as confirmatory feedback. Another example: Shiatsu givers will direct their *Hara* and thus the center of gravity directly at the region under treatment. The ideal direction will generate a feel of ease and non-deflected force. This feel progressively diminishes as one moves out into the “cone” surrounding the ideal vector, up to a threshold where it is lost (Figure 12).

To supply a last example, the quality of dyadic coupling can be monitored and shaped concerning the dynamic micro-interplay of forces. Practitioners report the optimal dyadic control state to involve another agency not hampering one's own and a sensory continuum with precise gradual scaling and without response delays or gaps. The loss of response continuity when a critical threshold is reached feels like a “barrier” or like something turning “rigid,” “dumb,” and “indifferent.” The perceived feedback signature (resistance, elasticity, etc.) begins to change qualitatively.

#### Further aspects of anticipation

**Figure 13:**
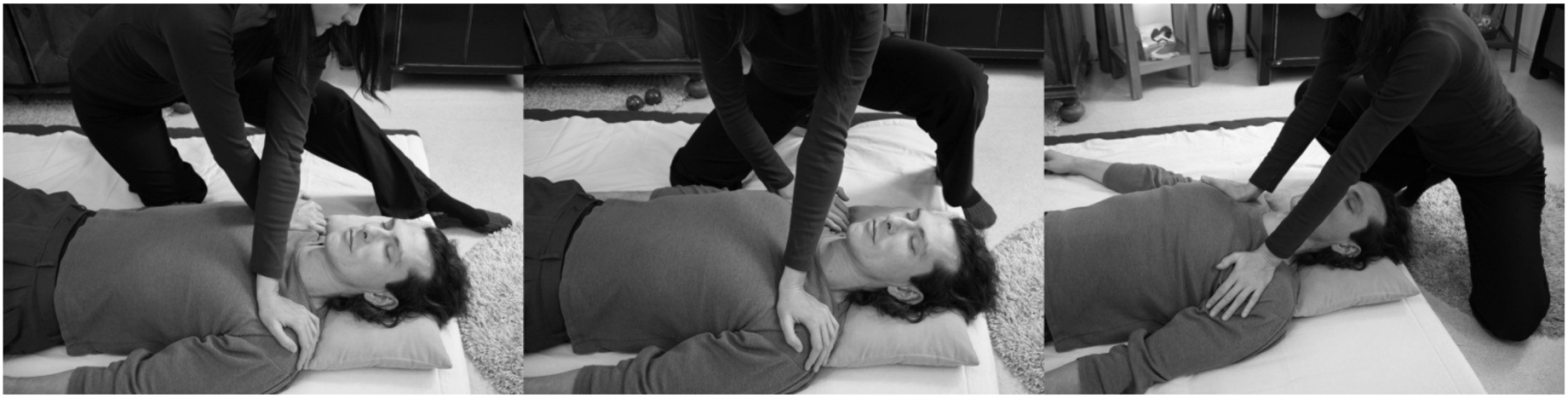
**End-position comfort through initial arm crossing (Shiatsu technique)**. As the practitioner moves around the client's body she aims at uninterrupted touch on the two shoulder interfaces. To ensure this, she begins in a rather awkward position she would not usually employ. As she rotates further, however, this guarantees an end-position where she comfortably can linger without having to lift the hands away for repositioning.

Mentally and motorically simulating outcomes in advance can be important for further reasons. In multi-phasic actions, anticipatory simulations allow an optimal build-up of the desired end-state, e.g., to maximize end-position comfort. A practitioner might start a grip technique with an awkward crossed arms position to get to the other side of the body in a continuous flow (Figure 13).

Anticipatory combined with enactive abilities allow practitioners to strategically exploit the structured temporal dynamics of coregulation. Thus, they can—actively or by waiting—bring about usable configurations at a remove (“I do X for the client to respond with Y, which lets me do Z”) and strategically generate affordances. Practitioners might, e.g., apply pressure for a muscle's release and hereby enable further diagnostic exploration of a ligament underneath. Knowing that—and how—one can adjust to the temporal dynamics expands the interaction possibilities (Section “Dynamic Solutions”). E.g., when pushing into the client's abdomen (*Hara*) Shiatsu experts can perform astonishingly deep stimulations because (a) they are alert to indicators of the client's pain threshold and possess salvaging strategies they dynamically apply before anything goes awry and (b) they fine-tune the action quality so it can be assimilated.

### Precalibrations

Bodywork apprenticeship instills general somatic dispositions that become a second nature in experts. Practitioners must employ comparatively unchanging bodily organizing principles to enable and sustain coregulation. They cultivate good somatic dispositions both of a task-specific and of a generally enabling sort (i.e., a permanent backdrop that guarantees fluid interaction as such).

At the session's outset the practitioner “slips into” a *habitus* which specifies permanent “dos” and “don'ts,” i.e., habits of posture, kinetic efficiency, motion range, attentional focus, muscle tension, and breathing. Thus, Feldenkrais and Shiatsu experts habitually initiate their own movements from their body centers, strive to continuously remain neutrally poised for action, etc. Habitus may become “naturalized” and, as Dreyfus and Dreyfus (1999) say, partly “hidden in the body.”

Habitualized action principles also furnish an efficient inter-body configuration: Shiatsu givers always direct power from their *Hara*. One image of this is to create a vector from the lower belly that is aligned with the focal zone of manual activity (Figure 12). Furthermore, bodyworkers in general refine their sensorimotor apparatus so as to become a good feedback environment for the client. They provide a “mirror,” “resonator,” and “amplifier” for the client's self-oscillation. Simultaneously, they contribute to the “unity of nervous systems” and create a backdrop of *we-intentionality*. These principles guarantee the rapport and constant information flow requisite for the build-up of all further dyadic synergies.

An accompanying *somatic mode of attention* (Csordas, 1993) specifies a host of epistemic strategies like using “soft eyes,” a defocused gaze. Active relaxation, a sense of balance, and minute proprioceptive awareness of breath, heart-rate and attentional states helps attune with the client. Organized “mental” interaction attitudes like being non-judgmental, unbiased, or contact ready reinforce this.

Overall, only educated bodies with properly constrained degrees of freedom can realize all the specific micro-skills we have discussed. We may think of this as intelligent presettings of muscle elasticity, joint radii, and so forth, which provide efficiency for specific techniques, but more fundamentally also enable interaction. With this, task management is partly offloaded to—albeit temporarily created—body structure (cf. Pfeifer and Bongard, 2007).

## Conceptual backdrops

Finally, we encounter hybrids of embodied-enactive and inferential skills in bodywork. Beyond their sensory and ideomotor routines practitioners throw genuinely representational skills into the mix (*contra* Hutto and Myin, 2012).

### Functional anatomy

One kind of representation in both disciplines is percept-near (*simulative, imaginative*): Functional anatomy models guide attention, as particular regions of the client's body are highlighted for exploration. Anatomical imagination also allows perception to become augmented through properties hidden beneath the skin into a rich multi-modal “image-percept.” Functional anatomy models can be thought of as dynamized text-book like images that simulate a limb in action. They are loaded with causal knowledge of the sort “when the shoulder blade is lifted the pull of the *Levator scapulae* muscle on the cervical spine is released.” Causality is based on observed perceptual covariance, which experienced practitioners can simulate off-line. Such models encapsulate biomechanical concepts like myofascial *tensegrity* (Ingber, 2008) or kinetic laws concerning levers, force transfer and deflection, etc. (Franklin, 1996). Thus, bodyworkers utilize precise images of functional body-part interrelations to actuate minute motion. Such simulative imagery defines afforded actions, maximal ranges of motion, etc., hence model-based perceptual inferences. Consequently, functional anatomy models enrich the interaction process via a kind of real-time embodied problem solving. The adduced information from functional anatomy is immediately merged into the sensory interaction dynamics for supporting diagnosis and action choice.

Since functional anatomy suggests causal contingencies between parts and wholes it inherently predisposes toward a systemic view of embodiment. Moreover, models are implemented in an inherently relational and holistic fashion. The way anatomy is taught to bodyworkers discourages a localistic and mechanistic course of action (as one might well see in more traditional biomedical quarters). This point further reinforces our claim that systemic relationality is inherent to bodywork (Section “How is Systemic Process Management Embodied?”).

### Abstract inferences

**Figure 14:**
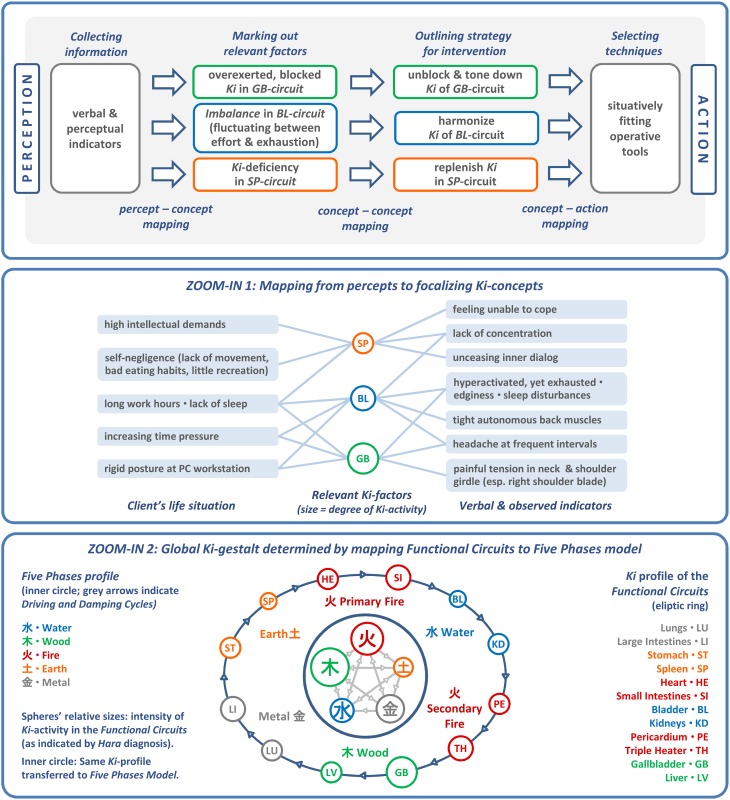
**Conceptual inferences within the perception-action loop**. The initially diagnosed state of the client's system shows a global imbalance of *Ki*. The practitioner singles out for treatment the three most disproportional aspects in the *Gallbladder* (GB), *Bladder* (BL), and *Spleen* (SP) *circuits*. The top panel encapsulates the idea that the diagnostic process runs via “hidden layers” at which conceptual mappings supervene in the practitioner's mind from diagnosis, via interpretation and strategy building to selecting operative tools. The center panel specifies the various informational sources brought to bear in the diagnostic phase, a mapping from many percepts to three focalizing concepts. The bottom panel goes into the details of the concept-to-concept mapping that is used to connect and further compress the global diagnostic gestalt from the twelve *Functional circuits* to the processual model of the *Five Phases*.

Beyond percept-near inferences, truly abstract representations are utilized in Zen Shiatsu. A sense of how complex these can be may be gleaned from the previously mentioned *Functional circuits* and *Five Phases* network models (Sections “Disciplinary Background and Goals” and “Synergetic Process Management”). For the treatment to begin, a perceptual gestalt is re-interpreted in theoretical terms (*Ki*-related concepts) and further disambiguated to suggest a course of action (Figure 14, top). The perception-action loop thus takes a “detour” by intermittently drawing on inferential processes.

- In a first step, information is integrated, including verbal reports, tactile indicators such as tight back muscles, and *Ki*-signatures perceived via palpation. This perceptual input now becomes enmeshed with corresponding concepts of *Kyo/Jitsu*—opposing attributions of *Ki* activity and saturation (Section “Probing the Attractor Landscape”). Sometimes working with regional *Kyo/Jitsu* profiles is enough for the practitioner to act. However, Zen Shiatsu also provides special procedures dedicated to synthesizing a global *Kyo-Jitsu* profile of *Ki* distribution across all *Functional circuits*. E.g., *Hara* diagnosis allows the practitioner to integratively map the verbal feedback and perceptual feedback gathered in respective diagnostic zones to the conceptualized logic of *Kyo/Jitsu* and *Functional circuits* (Figure 14, center and bottom).
- In sophisticated cases further concept-concept mappings may be added, such as mappings to the *Five Phases* model (Figure 14, bottom), a higher-level conceptual layer that summarizes how all twelve *Functional circuits* work together. This mapping serves complexity reduction. A dynamized version of the *Five Phases* model, the so-called *Driving and Damping Cycles*, encapsulates the functional interplay and possible transformations between *Functional circuits*. (Re-interpreting the basic conceptual model in terms of this processual rendering provides helpful inferences about the client's long-term system history, which we cannot explain here for space reasons.) Bear in mind, however, that not all of these resources need to be used at all times.
- At the stage of action planning, general praxeological principles need to be found that fit the diagnostic conceptual model. This type of inference either yields simple strategic routes of intervention such as “strengthen dominant *Kyo* and downtune compensatory *Jitsu*” or suggests multi-step strategies of synergy build-up. The Shiatsu vignette has illustrated this for three *Functional circuits*.
- For a final concretization of these strategies operative tools are selected (action concepts, control laws, sequence schemes, best practices). This happens with situated flexibility to optimally tailor the selected means, usually from a rich pool of options, to the client's emergent process dynamic.

To recap, inferences are utilized to link percepts and concepts, different levels of conceptual compression amongst each other, and the derived intervention strategies to the concrete action tool-box. We claim that various interdependent layers have to be coordinated in a complex parallel process that necessitates supplying inferences at the appropriate junctures while staying in touch with the client.

## Conclusion

Bodywork can be defined as continuous process management for somato-personal learning that respects moment-by-moment emergence. It is implemented via a tactile or otherwise sensory interface and follows a holistic philosophy of accompanying change in the client's systemic order. Under the master metaphor of achieving dynamic homeostatic balance, clients receive stimulations at multiple levels of their body system within the larger system of the stabilizing dyad. This triggers adaptive self-organization. The underlying idea, as in kindred systemic approaches, is to transform the attractor landscape for resilience in the longer run. Our aim has been to illustrate the types of process management strategies used:

- Bodyworkers combine perturbing and stabilizing stimuli to stimulate the client's system in a zone of proximal development.
- Through cumulative, at times recursive interventions a step-by-step reconfiguration of attractors takes hold in the client's system.
- The transformation process can be thought of as spreading activation in the somato-systemic architecture constituted by nested synergies and where the most encompassing level is a whole-body function.
- Bodyworkers ostensively operate within a more or less explicit image of this (hierarchical) architecture.

Our close analysis of vignettes illustrated that, as a basis for initiating action, practitioners probe the client's system to understand its order, especially imbalances. They (optionally) sketch-plan a macro-strategy for synergy build-up with greater or lesser projective reach, which is subsequently implemented through soft-assembly of synergies in response to the client's real-time feedback. While monitoring how synergies develop, practitioners can at every point customize strategies, switch from one operative tool to a better fitting one, or even take a different strategic route. The fact that systems are forever in flux and that this emergence must be respected necessitates systemic sensitivities, and these in turn require a flexible tool-box: Systemic constraints are involved in dynamically selecting and adapting operative tools—which are embedded in equally dynamic tactics and strategies.

Bodywork is unmistakably a “multi-skill.” A general mode of mind-body presence and a constant relational attentiveness need to be integrated in real-time with systemic awareness. These overarching factors need to be made co-extensive with the substrate of enactive micro-skills, which we have analyzed in detail (action repertoire, imagery, smart and dynamic perception, habitus, etc.). Although presence and attentive embodied rapport already accomplish a lot, bodywork is much more than benevolent touch: The numerous embodied micro-skills allow tailoring the intervention to each specific embodied dialogue's quality and dynamics. Hence, the way systemic awareness is augmented through embodied micro-skills commends bodywork in no small way. For therapeutic practice a hypothesis suggests itself: All other things being equal, skills gain leverage the greater their inter-enactive situatedness is (as indexed by continuity of monitoring and constancy of full-body presence). Situatedness in enactive terms cashes out as *dynamic immediacy* whereby the practitioner can respond with customized micro-interventions. To summarize, the “art of encounter” in bodywork consists in orchestrating multiple skill components, some at a more framing and some at a more situated level.

Understanding how the relatively abstract principles of DST are moored in the embodied-perceptual realm benefits greatly from the 1st person viewpoint of practitioners. Practical knowledge alone elucidates how systemic and embodied skills converge. Systems thinking left to its abstract devices will find it difficult to fully understand the micro-dynamics of bodywork. Micro-ethnographic analysis grounded in embodied, enactive, extended, and embedded cognitive science sheds light on these finer layers of the process. In summary, we hope that our case studies encourage *somatics* and therapy related areas to continue along these lines and further develop the interface between DST, embodiment theory, and their implementation in real-time interaction.

### Conflict of interest statement

The authors declare that the research was conducted in the absence of any commercial or financial relationships that could be construed as a potential conflict of interest.

## Footnotes

^1^Capitalized italics refer to concepts from subjective theories or models used by bodyworkers. All other italic expressions are used for highlighting analytic concepts when first introduced.

^2^*Meridians* are conceptualized as pathways along which *Ki* circulates through the whole body. They provide practitioners with a close access to *Ki* since it tends to accumulate there more tangibly than in other structures. (According to Eastern Medicine all living tissue is permeated with *Ki*.) Each of the 12 *Main meridians* is assigned to a *Functional circuit* and its respective tasks.

^3^Embodied “dialogs” have been investigated in infant development (Murray and Trevarthen, 1985; Stern, 1985; Fogel, 2006; Rączaszek-Leonardi et al., 2013) and adult communication (Fusaroli et al., 2014; Fusaroli and Tylén, 2015), as well as in minimal social behavior experiments (Auvray et al., 2009) and evolutionary robotics (Froese and Di Paolo, 2011). These approaches shed light on the dynamics of dyadic self-organization.

^4^Compare studies of we-intentionality (Schweikhard and Schmid, 2009), intercorporeity (Merleau-Ponty, 1964), super-individual *somatic modes of attention* (Csordas, 1993), the *embedded/extended body* (Clark, 2008), and embodied intersubjectivity more generally (Morganti et al., 2008; Semin and Cacioppo, 2008; Tomasello, 2008; Zlatev et al., 2008).

^5^In distilling order hierarchies from the data (Strunk, 2004), we restrict ourselves to the client's system, leaving order descriptions of the dyadic coupling for future study. In principle the inter-body coordination dynamics can be equally expressed through collective variables of synchronization, relative force, etc. (Oullier and Kelso, 2009; Schmidt et al., 2011; Dale et al., 2013). Examples include multi-agent synergies in sports (Passos et al., 2006; Bourbousson et al., 2010; Vilar et al., 2013), horseback riding (Peham et al., 2004; Lagarde et al., 2005), and teams (Gorman et al., 2010; Stevens and Gorman, 2011).

^6^To take a familiar experience, if we talk to someone in a frenzy in a markedly calm way (low intensity, deceleration), patting her arm while whispering (low intensity) to take it easy (redundancy) that person may well get more worked up. To be soothing we deployed every trick in the book—resulting in failure to pick up our friend from where she was! The overabundance of canonically “stabilizing” features unwittingly created an exaggerated contrast and amplified the disorder.

^7^Anticipatory assessments are required to estimate which critical fluctuations partake of a sound developmental trajectory and which ones put the system's integrity at risk.

^8^The gestalt-building procedure is reminiscent of idiographic models in psychotherapy research (Strunk and Schiepek, 2006, p. 176ff; Haken and Schiepek, 2010, p. 432).

^9^The FI related quote by Rywerant (2003, p. 82) fits this idea: “While exploring the range of movement the teacher first makes sure that the habitual sector of that range is established as well-known and familiar. This middle-part of the range of movement is usually somewhere around the neutral point of least effort. Only with the acknowledgement of this neutral area as safe can one address oneself to the pupil's upper level of control.”
